# Understanding the degradation of Ag_2_Cu_2_O_3_ electrocatalysts for CO_2_ reduction†

**DOI:** 10.1039/d5na00328h

**Published:** 2025-08-14

**Authors:** N. Vorlaufer, J. Josten, A. Hutzler, C. A. Macauley, N. Martić, M. Weiser, G. Schmid, K. J. J. Mayrhofer, P. Felfer

**Affiliations:** a Institute I, Materials Science & Engineering Department, Friedrich-Alexander-Universität, Erlangen-Nürnberg (FAU) Martensstraße 5 91058 Erlangen Germany nora.vorlaufer@fau.de; b Helmholtz Institute Erlangen-Nürnberg for Renewable Energy (HI ERN) Egerlandstraße 3 91058 Erlangen Germany; c Interdisciplinary Center for Nanostructured Films (IZNF) Cauerstraße 3 91058 Erlangen Germany; d GmbH Siemens Energy Global GmbH & Co. KG, SE TI SES PRM CT AEM Freyeslebenstr. 1 91058 Erlangen Germany nemanja.martic@siemens-energy.com

## Abstract

Recently, a mixed-metal oxide with a paramelaconite-type crystal structure (Ag_2_Cu_2_O_3_) has been investigated as a promising catalyst for electrochemical reduction of CO_2_ and CO. The catalyst operates with a reasonable overpotential and good selectivity. However, during its utilization, the catalyst experiences a degradation in conversion efficiency, thus limiting its potential in industrial application. This has so far been attributed to the unstable nature of the crystal structure, which tends to partition into metallic copper and silver. In this study, we characterized this decomposition using atom probe tomography and analytical electron microscopy. We found this decomposition to take place also under an electron beam without any ongoing reaction conditions. We also found that dissolution mechanisms must play a role in the degradation of the catalyst. This is deduced from the existence of nanostructures which only form during catalyst operation and are comprised of copper and potassium, the latter of which stems from the electrolyte. The composition of these nanostructures was confirmed using an atom probe.

## Abstract 2

In this work, we characterized the degradation behavior of a CO_2_ catalyst and show the influence of characterization methods (mainly electron microscopy) on the catalyst. We show the formation of a K enriched phase throughout the reaction.

## Graphical abstract

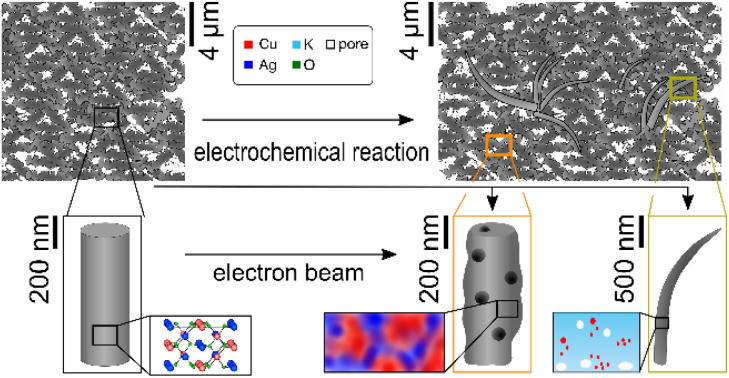

## Introduction

The conversion of CO_2_ or CO into high value chemicals through CO_2_/CO electrolysis by using renewable energy is a promising approach to create a sustainable circular carbon economy.^1^ In recent publications silver–copper catalysts and other bimetallic alloys (tandem catalysts) have received considerable attention for CO_2_ electrolysis.^2–5^ The intention is to enable the direct reaction of CO_2_ to C_2+_-products *via* a spill-over reaction. Spill-over reactions at silver–copper (Ag–Cu) catalysts aim to directly catalyze the reaction from CO_2_ molecules to C_2+_-products, by firstly reducing CO_2_ to CO at the Ag active sites. This is then followed by subsequent reduction of CO to C_2+_ products at the neighboring Cu active sites.^6–9^

Regarding Ag–Cu tandem catalysts, various groups^2–5^ discovered several differing factors to distinctly influence the reaction. Zhang *et al.*^2^ investigated Cu–Ag core–shell particles. They found the thickness of the Ag shell to have a major influence on the selectivity of C_2+_-products. Iyengar *et al.*^3^ combined the investigation of Ag–Cu tandem catalysts with facet dependent selectivity and found the combination of facet tuning and a tandem catalyst to further influence the reaction. In another study Iyengar *et al.*^4^ created a model catalyst system of Ag nanospheres and Cu nanocubes. They linked an increasing ethanol/ethylene ratio to the edge contribution of the nanocubes. Liu *et al.*^5^ created pyramidic tandem catalysts. They found a high faradaic efficiency (FE) for C_2+_-products and a low FE for CO. However, the authors proposed that spill-over cannot solely account for the observed reaction. This debate underlines the importance of understanding the dynamic processes during the electrochemical reaction. It is of high interest to identify the structure–property-relationship to stabilize and improve the catalytic reaction.

In this study, we elucidate the local distribution of Ag and Cu by a microscopic investigation of the catalytic particles before and after the reaction. For resolving local variations down to the sub-nano scale, we combined atom probe tomography (APT) and transmission electron microscopy (TEM), which are both investigation methods with near atomic resolution.

For APT, a tip-shaped specimen with a tip apex radius around 100 nm (ref. 10) is required. The specimen is cooled down to a temperature below 80 K. By applying high voltage pulses up to 15 kV (ref. 10) atoms are field ionized individually, evaporated from the tip apex and accelerated towards the detector. These field-evaporated ions can be subsequently detected and their initial position reconstructed. The chemical nature of the evaporated ions can be determined using the characteristic mass-to-charge states of isotopes based on the time-of-flight measurement. This also includes the identification of light elements, which is especially important for this study. With APT, a three dimensional reconstruction of the initial tip can be created, enabling the detailed investigation of nanostructures.^10–12^

The complementary use of scanning transmission electron microscopy combined with energy dispersive X-ray spectroscopy (STEM-EDXS) and APT proved helpful to reveal structure–property relationships in various studies.^13–18^ With TEM the direct observation of samples with a diameter of around 100 nm (ref. 19) is possible, which enables the investigation of the particles without the need for further preparation steps. Additionally, TEM is not a destructive measurement method.^20^

**Fig. 1:**
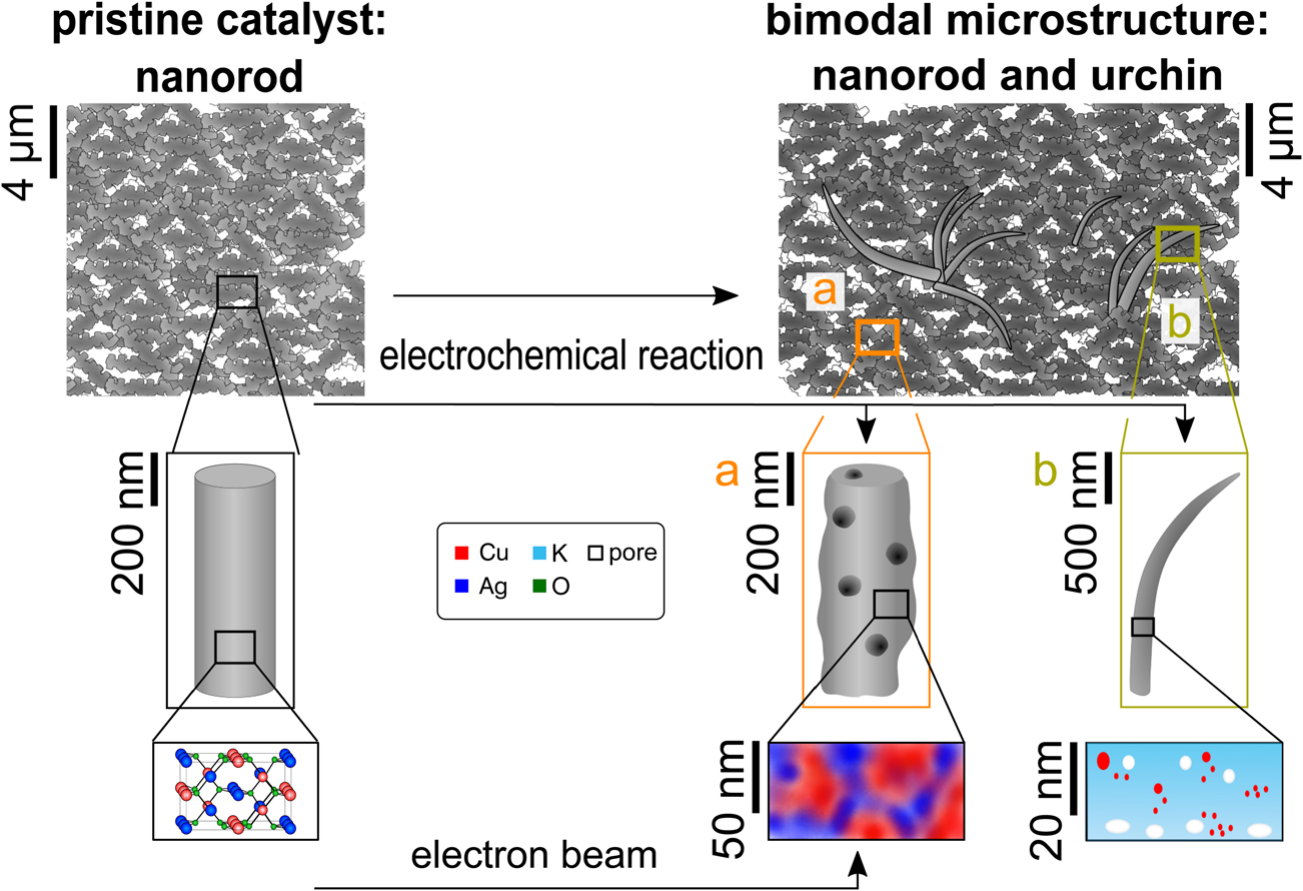
Hierarchical schematic of the catalyst evolution due to the electrolysis reaction and the influence of the electron beam on the pristine particles. On the left hand side, the pristine catalyst, the Ag_2_Cu_2_O_3_ powder, is displayed in various resolutions. The right hand side displays the evolution in different magnifications due to either the electron beam or the electrochemical reaction. (a) The evolution of the nanorods, (b) the newly evolving “urchin” microstructure.

We study Ag_2_Cu_2_O_3_ nanorods, which were used by Martić *et al.*^21^ in CO_2_ electrolysis reactions, using both APT and TEM. They exhibit promising properties concerning operation at a reasonable overpotential and selectivity. Our results offer a detailed picture of the Ag_2_Cu_2_O_3_ catalysts' microstructure before and after the electrochemical reaction, describing the evolution of a new microstructure during the electrochemical reaction. Additionally, we describe the probing damage of STEM and scanning electron microscopy (SEM) investigation methods on the nanoparticles. The investigation method can actively change the structure, morphology, and composition of the investigated catalyst. A schematic overview containing our findings is pictured in Fig. 1.

## Materials and methods

The catalyst Ag_2_Cu_2_O_3_ powder was synthesized at room-temperature by alkaline coprecipitation of a mixed solution of copper(ii) nitrate and silver nitrate, as described by Martić *et al.*^21^ The electrochemical CO_2_ reduction experiments were performed in a flow reactor (Micro Flow Cell, ElectroCell) which utilizes a three-electrode set-up. The electrolysis cell consists of one gas and two electrolyte flow channels. At the cathode side, the gas flow channel is separated from the catholyte by a gas diffusion electrode (GDE) which served as the cathode. The GDE was fabricated by spray-coating Ag_2_Cu_2_O_3_ nanorods (loading 1.5 mg cm^−2^) on top of a commericially available GDL H23 C2 (Freudenberg SE, Germany). A commercially available anion-exchange ionomer from Dioxide Materials (Sustainion XA-9) was used. At the anode side, oxygen is formed through the oxygen evolution reaction and extracted from the reactor together with the anolyte. A solid titanium plate coated with IrO_*x*_ (ElectroCell) and Ag/AgCl (3 M KCl) were used as anode and reference electrode, respectively. The two electrolyte flow channels were filled with 1 M KHCO_3_ (catholyte and anolyte) and separated by an anion-exchange membrane (Fumasep, FAB-PK-130) which serves as an electrode separator. The experiment was carried out under a constant current density of 300 mA cm^−2^ for 48 h with a corresponding potential measured at the cathode of approx. −1.3 V *vs.* RHE.

Ambient air heat treatments of the pristine particles were performed in an air circulation furnace (Arnold Schröder Industrieöfen GmbH, Germany) at 150 °C and 300 °C respectively.

Imaging with an SEM was performed using an XB540 FIB-SEM (Carl Zeiss Microscopy, Germany). For the investigation of the electron beam influence on the nanoparticles the imaging settings varied. The images were taken with an acceleration voltage of 3 kV or 15 kV, while the beam currents were varied between 500 pA and 4 nA. Additional electron microscopy characterization was carried out with a Talos F200i (S)TEM (Thermo Fisher Scientific, USA) equipped with a Dual Bruker XFlash 6T-100 EDXS detector. The microscope was operated at a primary electron energy of 200 keV and a probe current of 155 pA in high-angle annular dark field STEM mode (HAADF-STEM).

**Fig. 2:**
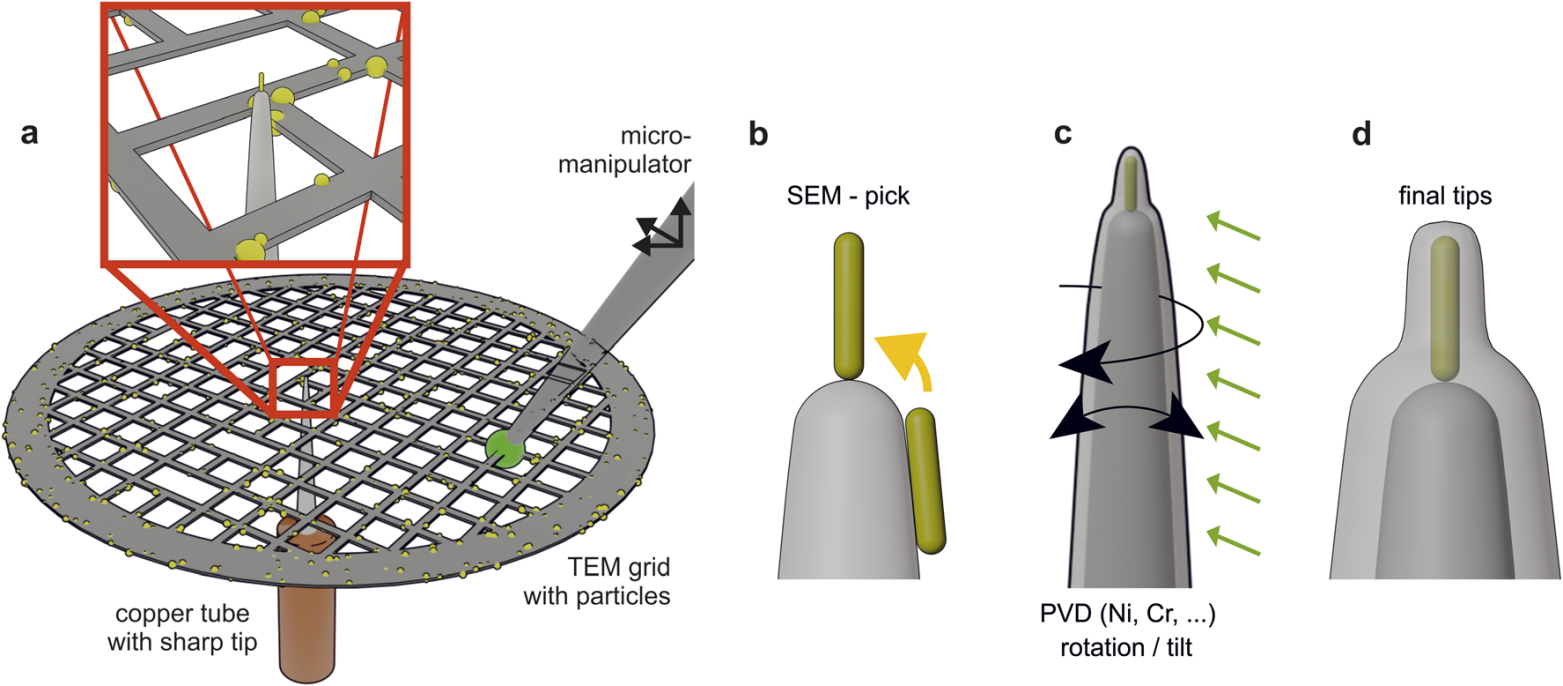
Illustration of the pick-and-coat method for the preparation of nanoparticles for the atom probe. (a) The particles dispersed on the grid. (b) The approach for the correct positioning of the particle, before it gets coated (c) to improve the stability of the APT measurement. (d) The final tip. Scheme modified from Josten and Felfer.^22^

To incorporate the nanoparticles inside an APT tip, we employed the pick-and-coat sample preparation.^22^ The nanoparticles are transferred from a lacey carbon TEM grid to the apex of substrate tips, using micromanipulation (see Fig. 2). The compound of nanoparticle and substrate tip is evenly coated using PVD. A more detailed description of the method can be found elsewhere.^22^

The tungsten support tips for the APT investigation were prepared by electropolishing tungsten wire in 5% sodium hydroxide (NaOH) aqueous solution.^23^ The applied voltage was 5 V.

The nanoparticles are put on TEM grids, which are equipped with lacey carbon layers (purchased from Plano GmbH, Germany). The pristine particle powder was dispersed on top of the grid. The electrochemically deployed catalyst particles were manually removed from the GDE with a spatula. The resulting powder was put on the TEM grid the same way as the pristine particles. After clamping the TEM-grid with a copper tube, the assembly was mounted on a MM3A-EM micromanipulator (Kleindiek Nanotechnik GmbH, Germany) inside of the SEM chamber.

For the SEM part of the pick and coat method, we used an acceleration voltage of 3 kV and a beam current of 1 nA. The tungsten tip on the SEM stage is movable separately from the particle grid. By scratching the desired nanoparticles off the TEM grid, particles are transferred to the tip and subsequently positioned on top of the tip, as shown in Fig. 2. To prepare the final APT tip a coating is applied utilizing a custom-built PVD setup. The used target materials are nickel and chromium, both supplied by HMW-Hauner GmbH & Co KG, Germany. The APT measurements were carried out in a reflectron LEAP 4000X HR (CAMECA, France) in laser pulsing mode. The temperature was set to 55 K, the laser pulse energy to around 70 pJ and the pulse rate was set to be suitable for heavy ions to be detected (100–125 kHz). Reconstruction and interpretation was done using IVAS 3.6.8 (CAMECA, France) and afterwards MATLAB (MathWorks, USA) scripts that were developed in-house and can be accessed *via* github (https://github.com/peterfelfer/Atom-Probe-Toolbox).

## Results

In this paragraph we describe the results of the various characterization methods. After the electrochemical classification, the evaluation of the various microscopy techniques, namely SEM, TEM and APT, is shown.

### Electrochemical results

**Fig. 3:**
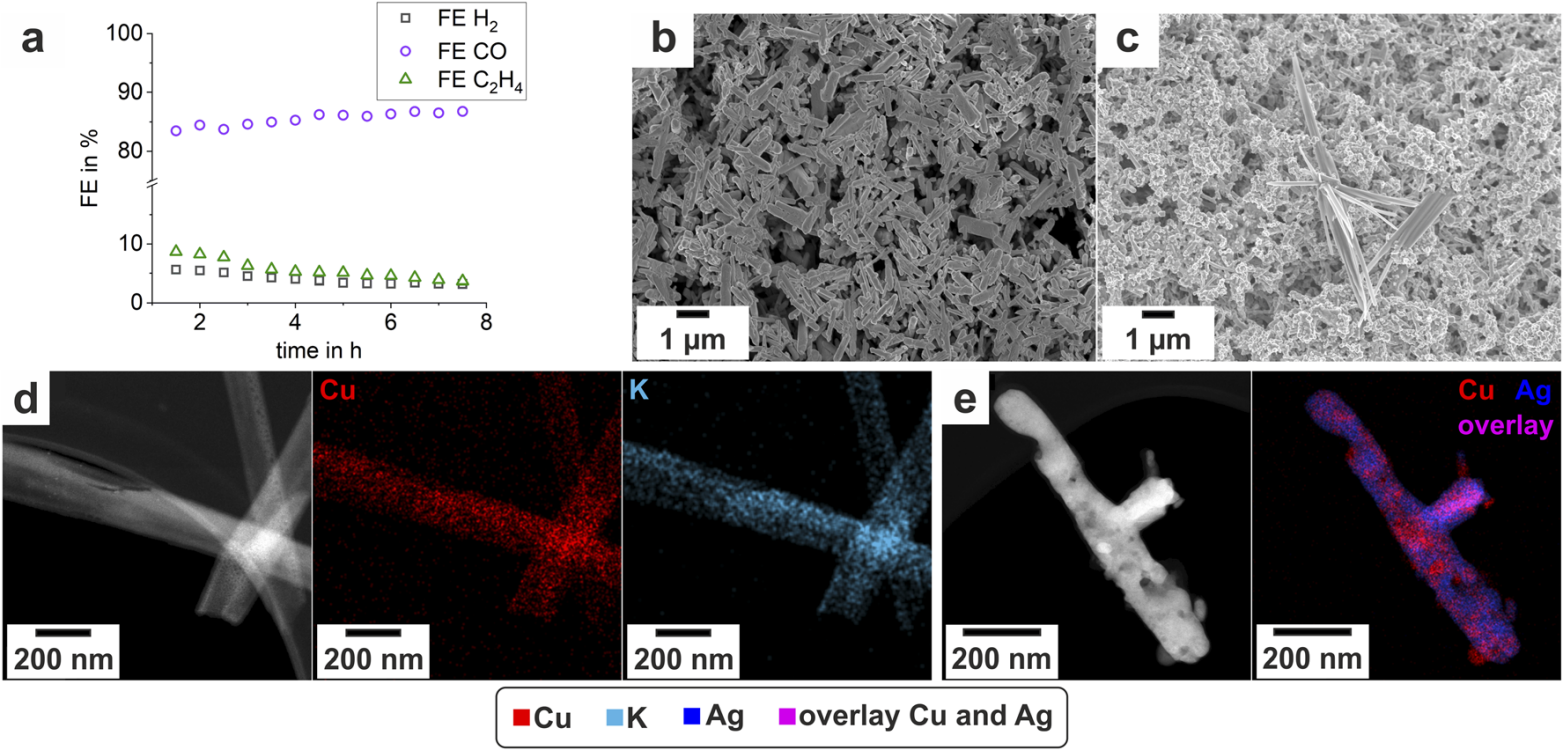
(a) The evolution of the FE as an indication for product evolution for the first 8 h of the reaction. The reaction was carried out with a current density of 300 mA cm^−2^ and a corresponding potential measured at the cathode of approx. −1.3 V *vs.* RHE. Further catalytic data can be found in ref. 21. (b) The pristine nanoparticles and (c) the catalyst after the employment in the electrochemical cell. (d) and (e) STEM-EDXS images of electrochemical deployed particles. The urchin (d) mainly consists of Cu and K, while the main components in the residual nanorod (e) are Cu and Ag.

During the entire length of the experiment, gas products were continuously monitored utilizing an online gas chromatograph. This allowed us to demonstrate the trend of the detected reaction gas products, with the FE plotted over reaction time in Fig. 3a. The FE was calculated with the following formula:
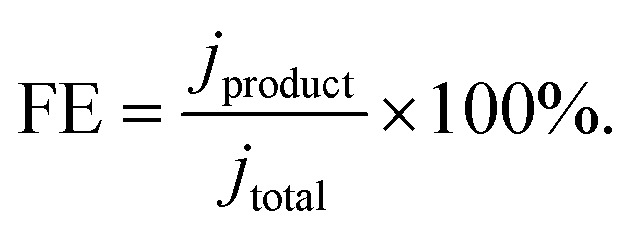


As can be seen from the results, the catalyst successfully suppresses the unwanted, parasitic HER with FE H_2_ being kept below 5% and steadily decreasing with time. When analyzing the FE for CO and C_2_H_4_, it is obvious that there is a shift in selectivity as the electrolysis progresses. With time, the catalyst becomes more selective towards reducing CO_2_ to CO, while the FE for C_2_H_4_ simultaneously drops. In their publication, Martić *et al.*^21^ have argued that the shift in selectivity is related to the catalysts' surface reconstruction where the catalyst loses its initial structure and the surface becomes more saturated with Ag. Hence, the formation of CO intensifies due to a larger number of exposed Ag-rich surface active sites, while on the other hand, the selectivity for ethylene drops due to a decreasing concentration of the original Cu active sites at the surface.

### SEM results

The pristine particles exhibit a rod structure but show no homogeneous size distribution, see Fig. 3b. XRD measurements from a previous publication^21^ proved the nanorods to be a Ag–Cu mixed-metal oxide Ag_2_Cu_2_O_3_ with a paramelaconite type crystal structure.

When the particles are deployed in an electrochemical reaction the morphology alters drastically (comparing Fig. 3b to c). Firstly, the nanorods evolve a pimpled surface, while their average size decreases. Additionally, a new type of particle microstructure evolves. The formation of elongated “urchin-structures” on the surface of the GDE is depicted in Fig. 3c. EDXS spots indicate that this precipitate is Cu rich. Also, the intensity of the potassium (K) peak is higher, compared to EDXS spots on the nanorods (see Fig. 1 ESI†). In the following, when discussing the electrochemical deployed catalyst, the nanorods will be referred to as “residual nanorods” and the newly evolved structure as “urchin-structures”.

**Fig. 4:**
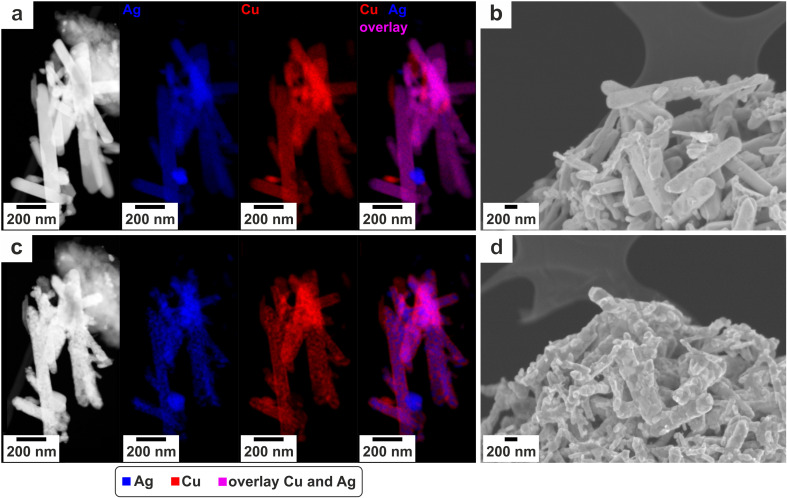
Influence of electron irradiation on the pristine nanorods. (a) and (b) Pristine nanorods in the TEM and the SEM, respectively. (c) and (d) The evolution of the nanorods after consecutive illumination with the electron beam. The transformation in (d) was induced with an electron beam of 15 kV and 3 nA.

While examining the pristine particles in the SEM the morphology of the particles changes. This is shown in Fig. 4b and d. The altered particles are covered in small spheres. Those spheres are similar to the pimples that occur on the electrochemically deployed catalyst particles. They occur rapidly during the examination with the electron probe of the SEM and efforts to effectively reduce them is ongoing.

By varying the parameters of the electron probe, the driving force for the artificial-aging of the pristine particles was investigated. The different outcomes can be seen in Fig. 2, ESI.† The change of the microstructure under the SEM electron beam appears to be dependent on the applied current and the applied voltage. The higher the voltage and the current, the faster the spheres occur and coalesce. In the most extreme cases the spheres develop within one single frame. In such cases after short irradiation times the spheres partially move over the surface and the whole surface is rapidly covered. Additionally, the evolution of the microstructure is dependent on the substrate. When placing the particles on a carbon-adhesive, which commonly used to mount SEM samples, the alteration does not occur.

The same trend applies to heating the nanoparticles. After heating the particles in a furnace in ambient air to 150 °C for an hour, the microstructure of the calcined particles does not vary from the pristine particles, while the heating to 300 °C induces a clear change in morphology (see Fig. 3, ESI†). Fig. 4, ESI† shows that a morphology change of the microstructure also occurs while imaging the urchin-structures. They are sensitive to beam damage and are wrinkling during the examination with the electron beam.

### STEM results

**Fig. 5:**
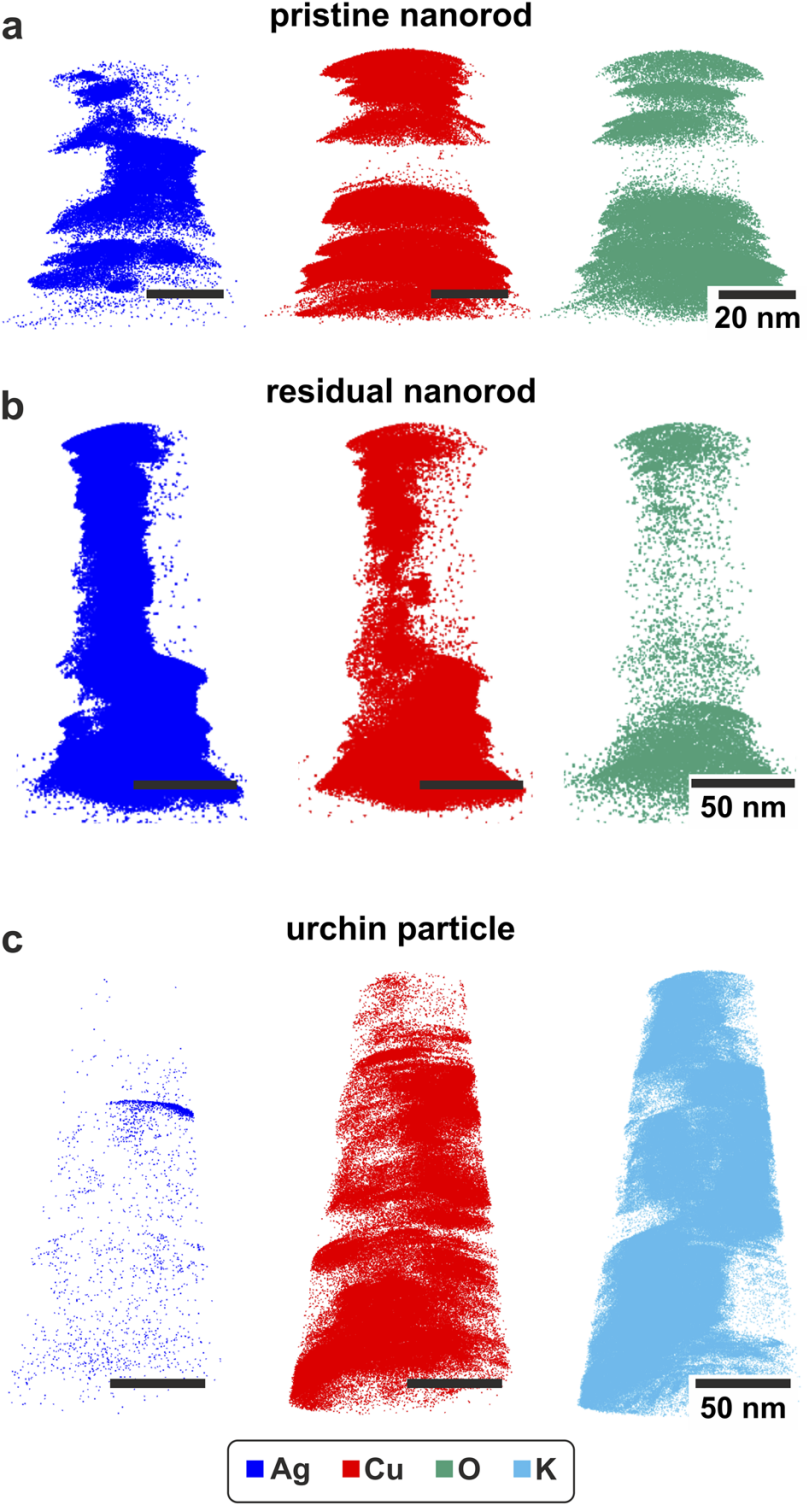
APT reconstruction of the measured particles. (a) The reconstruction of the pristine particle that consists of partial demixed Ag and Cu. The same goes for the residue nanorod in (b). The urchin structure in (c) consists of a 3 D-structure made of K and Cu, while the Ag fraction is low. The corresponding mass spectra are shown in Fig. 11, ESI.†

STEM EDX spectrum images of the pristine particles are displayed in Fig. 4a. During the examination with the TEM an alteration of the pristine particles takes place, though not as fast and as clear as in the electron beam of the SEM. It takes several minutes of irradiation with the STEM probe to evoke an apparent alteration. The evolution is depicted in the spectrum image in Fig. 4a and c. Interestingly the morphology change is accompanied by a demixing of Ag and Cu. When comparing the peak intensities of Ag to Cu the ratio remains constant, being 1 : 1. In Ag-rich regions no residual oxygen was detected (see Fig. 5, ESI†).

Also, for the residual nanorods a change of the microstructure can be observed after illumination with the electron beam (see Fig. 6, ESI†), even though the change is not as rapid and as clear as for the pristine nanorods. Concerning the elemental evaluation of the electrochemically deployed particles, an EDXS STEM map of a residual nanorod and an urchin can be seen in Fig. 3d and e. The urchin-structure mainly consists of K and Cu. Micrographs with higher magnifications demonstrate small agglomerations with a diameter below 4 nm (see Fig. 7, ESI†). The morphology of those particles changes during the illumination of the frame and additionally pores evolve inside the structure.

The HAADF-STEM images (Fig. 3e) of the residual nanorods reveal pores within the nanorods, while the EDX spectrum image indicates an inhomogeneous distribution of Ag and Cu. The elements show a pattering morphology. At higher magnifications (Fig. 8, ESI†) a light cloud surrounds the nanorods' border. EDXS scans strongly indicate this region consists of pure Cu.

In the enriched Cu-region oxygen is also verified (Fig. 8 ESI†). *In situ* XRD measurements by Martić *et al.*^21^ support our EDXS measurements. They determined the surface of the electrochemical deployed catalyst to consist of metallic Ag and Cu. The measured oxygen, as in our case, is attributed to the oxidation of Cu in air.

### APT results

Using the pick and coat method we were able to measure a pristine particle in the atom probe, see Fig. 5a. To evaluate the measured data, regions of interest (ROIs) were selected in the dataset, as indicated in Fig. 9, ESI.† For each ROI a 2D concentration map for Ag and Cu atoms was created. Histograms of each concentration map, which indicate potential demixing of Ag and Cu, are shown in Fig. 9, ESI.† The *x*-axis represents the ratio of the number of Ag atoms to the sum of Ag and Cu atoms, while the *y*-axis displays the amount of voxels with a base area of 1 × 1 nm with the respective composition. The evaluation of the APT data shows a heterogeneous structure of the measured pristine particle. Except for one ROI, which contains a considerable amount of Ag rich voxels, the other two ROIs consist mostly of pure Cu. In the Cu region the presence of oxygen was verified, while the Ag rich region shows no oxygen content at all (see Fig. 5a).

Concerning the preparation of the electrochemically deployed particles *via* the pick and coat method, special attention was paid towards targeting both particle-types. The particles that were selected for detailed analysis can be seen in Fig. 10, ESI.† One corresponds to the residual nanorod, and the other selected particle exhibits a varying morphology resembling the urchin-structures that can be seen in SEM images (see Fig. 3c). Thus, we assume this small particle was a part of an urchin-structure that was broken while preparing the TEM grid for the pick and coat process. The interpretation of the APT data (see Fig. 9, ESI†) was executed the same way as for the pristine particle.

Regarding the residual nanorod in Fig. 5b it is obvious that the main part of the particle is Ag rich, namely approximately 92 at% (measured atoms, concentration and standard deviation stated in Fig. 10, ESI†), the measurement also contains a few K ions.

Concerning the histograms of the residual nanoparticles in Fig. 9, ESI,† most of the voxels contain pure Ag. Only the lower regions of the particle show a mixing of Ag and Cu. Also, those mixed regions exhibit a higher Ag to Cu ratio.

The second APT dataset from the urchin-structure in Fig. 5c draws a different picture. Around 75% of the measured dataset consists of K. Another primary content is Cu, while only a small fraction of the dataset, 0.4 at%, consists of Ag. Considering the 2D-concentration maps, the K and Cu rich regions exist side by side and are not mixed (Fig. 9, ESI†).

## Discussion

### Change of the microstructure: integration of a new microstructure and redistribution of nanorods

Concerning the urchin-structures, the APT data demonstrate a three-dimensional network of Cu and K ions. The APT data prove the K information imaged in the STEM-EDX spectrum image is not a preparation effect of the particles. Both measurements show Cu and K nano-islands co-existing in a patterned structure (see Fig. 7, ESI and Fig. 9, ESI†). A patterned surface of the urchin-structures is also observed in the SEM investigations, in Fig. 4, ESI.† This surface pattering possibly corresponds to the Cu/K element patterning observed in APT and spectrum imaging. The patterns change in size and morphology during the electron beam imaging process, as discussed in more detail below.

As both techniques, the STEM EDXS and the sample preparation for the APT specimens, include imaging with the electron beam, the initial state of the urchin-structure, without electron irradiation, cannot be defined unambiguously. Two options are conceivable: (1) Cu and K are present in the form of a solid solution, (2) Cu and K are present as sub-nano domains that enlarge during the electron irradiation. Known K–Cu compound are K-cuprates (KCuO_2_).^24^ Since we did not observe oxygen in our atom probe dataset, we assume our investigated compound is not related to K-cuprates. Also, we are not aware of any existent K–Cu phase diagram.^25^ However, phase diagrams describe the thermodynamic equilibrium of the bulk material, so the existence of a K–Cu solid solution of a nanostructure cannot be excluded. To finally clarify the initial state of Cu and K inside the urchin-structures, further investigations are required.

Interestingly, neither the STEM EDXS nor the APT measurements show a carbon precipitation (see Fig. 7, ESI†). Thus, the urchin-structure development cannot be attributed to carbonate precipitation from the electrolyte, as described in various studies.^26–28^ Apart from the three-dimensional K precipitation inside the urchin, another interesting fact is the Cu-content of roughly 30 at% (see the table of Fig. 10, ESI†), indicating that Cu from the pristine nanorods must play a role in the formation of the urchin-structure. Sassenburg *et al.*^29^ found Cu to reorganize into new needle-like structures, which visually resemble the urchin structures reported in this study, thus underlying our finding of the newly evolved structure being Cu-enriched.

The influence of CO_2_ operation conditions on Cu is described in the literature by several authors.^30–35^ On one hand, rearrangement processes of copper as the reason for Cu morphology change are discussed extensively in the literature. Huang *et al.*^31^ describe a morphology change for Cu particles that visually resembles the evolution of the nanorods in our experiment. Due to low applied potentials and the fact that no Cu was detected in the electrolyte they exclude a dissolution process. They find the adsorption of CO and H in combination with negative potentials to play a key role in the rearrangement process. They also encounter a decrease in the FE of ethylene, connecting this phenomenon with the rearrangement of Cu and an accompanying change of the surface planes. Also, other researchers describe a rearrangement process^32–35^ and do not consider a possible dissolution of Cu.

On the other hand, dissolution processes are also discussed as the decisive reason for the Cu morphology alteration. Speck *et al.*^30^ found that Cu dissolves when applying a positive potential. The dissolution process is dependent on the pH value. A low dissolution rate was found to exist around 8/9 pH, with the dissolution rate increasing with higher pH values. The studies of Vavra *et al.*^36^ and Popovic *et al.*^37^ strongly indicate a factor for the morphology change to be dissolution of Cu-oxide at the open circuit potential step of the process, at which Cu dissolves into the electrolyte. Also, Raaijman *et al.*^38^ eventually state that in their identical location investigation, a morphology change under CO_2_ reduction representative conditions was only seen for Cu-oxide surfaces, but not for metallic Cu surfaces. They attribute the Cu morphology change described by other investigators to accidental Cu oxidation.

Wilde *et al.*^39^ however describe Cu-dissolution in their *in situ* TEM study. Chen *et al.*^40^ also infer that the observed separation of Cu and Ag evolved due to Cu-migrating and detaching from the catalyst surface. Finally, Vavra *et al.*^41^ showed that Cu detaches in the form of [CuCO]^+^ complexes, before redepositing as metallic Cu. Also, Tomc *et al.*^42^ observe a dynamic equilibrium of dissolution and redeposition of Cu throughout the electrochemical CO_2_ reduction.

So, we can conclude that Cu ions are temporarily present in solution, before they directly redeposit with K in the form of the urchin-structures. Transferring this to our experiment, we see that the urchin-structures are a completely new microstructure that forms during the electrochemical experiment. The size of the urchins, with a length usually ranging between 5 μm and 20 μm, exceeds the size of the pristine nanorods, which are in the size range of 0.5 μm to 2.5 μm.^21^ So, a rearrangement process cannot be taken as the sole explanation for the formation of the urchin-structure and is unlikely. We conclude that our investigations indicate a combination of Cu-dissolution and subsequent redeposition. Whether the dissolution of Cu-sites occurs in the context of the initial Cu-oxide structure, or after the reduction to Cu, cannot be concluded from the results of this study.

The demixed regions of Ag and Cu also hint in the direction of Cu-dissolution. The TEM investigation in Fig. 8, ESI† shows decomposition of Cu rich regions. We observe pores and Cu-clouds around the particles. In general, the STEM investigations show porosity of the residual nanorods (see Fig. 3e). The Cu clouds represent an extension of the surface, but commonly, rearrangement processes, like *e.g.*, sintering, are accompanied by reduction of surface area.^43^ Also, the Cu content determined for the APT dataset (Fig. 10, ESI†) was rather low. So those four points: former studies indicating Cu-oxide or Cu dissolution,^36–42^ the evolution of a huge new microstructure, the generation of pores in the residual nanorods during the process and the increasing surface area, strongly point towards dissolution rather than a rearrangement process.

The dissolution process, the change of morphology of the particles and the integration of a new microstructure prove that the catalyst has a very dynamic surface during the reaction.

The new urchin-structures have, due to their huge size, a lower surface-to-volume fraction compared to the nanorods. As a major content of the urchin-structure is Cu, the urchins thus withdraw a significant amount of Cu atoms from being available at the surface. The K in the urchin-structure additionally blocks Cu sites from participating in the reaction. The even distribution of the urchin-structure on the GDE implies that available Cu sites are decreasing all over the catalyst, thus significantly influencing the microenvironment around the catalyst particles. Additionally, the observed morphology change of the nanorods, see Fig. 3b and c, and the Cu-clouds around the nanorods (as seen in the TEM images in Fig. 8, ESI†) highlight the dynamic catalyst surface throughout the reaction. This further indicates that the microenvironment of the catalyst particles is non-static, possibly influencing the selectivity and activity of the catalyst.

Other literature studies on Ag–Cu tandem catalysts highlight the importance of the spatial vicinity of Ag and Cu to enable the spill-over effect and thereby the generation of C_2+_-products.^5,44^ When correlating the electrochemical data (Fig. 3a) to the described evolution of nanorods, the “striped” Ag–Cu surface, as indicated in Fig. 3e, should be ideal for spill-over reactions. However, the evolution of the Cu clouds is accompanied with an extended Cu surface and thereby less adjacent Ag–Cu interfaces. Additionally, the urchin-structures lower the Cu sites that are available in total. Also, the Cu clouds do not have a defined, ordered structure. Thus, desirable surface planes, like [100],^45,46^ for the conversion of C_2+_-products are not stable on the surface. This seems to influence the product selectivity in a way that fewer C_2+_-products are formed, what can be seen in the trend of the electrochemical data.

The dynamic microenvironment of the particle surface in general makes it hard to perform a controlled reaction with targeted conversions and reactions.

### Influence of the electron beam

#### Pristine particles

The microstructure of the pristine nanorods changes during the imaging with SEM (Fig. 4b and d) and TEM (Fig. 4a and c). The STEM EDX spectrum images of the artificially aged particles show Ag and Cu dealloying and the size of the Ag spheres increasing, Fig. 4c. In the Ag-region no oxygen was measured (Fig. 5, ESI†). This indicates that the electron beam triggers a reduction reaction of the Ag-oxide-bonds to Ag. Additionally, the microscopic pattern of the distribution of Ag and Cu is similar, when comparing the residual nanorod after the electrochemical reaction and the artificially-aged nanorod. This is visible in the micrographs of the artificially-aged and the residual-nanorods in Fig. 4c and 3e.

The mobility of Ag under the influence of the electron beam is described in several publications,^47–51^ but none provides a final explanation for the Ag movement. To deduce the driving force for the artificial aging with the electron beam in our case, the nanorods were calcined. No change of the structure was observed at 150 °C, but the temperature of 300 °C triggered a change of morphology. This is not surprising as 300 °C is close to the thermal decomposition temperature of Ag-oxide to Ag in air, which is around 350 °C.^52^ The reduction equation can be described as follows:^52,53^Ag_2_O (s) → 2Ag (s) + 0.5O_2_ (g).

Nanoparticles often exhibit different mechanical properties compared to the bulk material. For example, the so-called melting-point depression describes the phenomenon of the reduction of the melting point with decreasing particle size.^54–56^ Taking this into account, the assumption of thermal decomposition already initiating at 300 °C is realistic. The macroscopic morphology of the calcined nanorods as can be seen in the SEM images in Fig. 3, ESI† resembles the morphology of the residual nanorods that were deployed in the catalytic reaction.

This leads to the hypothesis that in all three situations (calcined nanoparticles, artificial aging with electron irradiation and electrolysis reaction) a similar reaction, the reduction of Ag-oxide, takes place.

The thermal decomposition of Ag-oxide is a known phenomenon as described above. To our knowledge, the reduction of Ag-oxide due to an electron beam is not described in the literature. When discussing the heat influence of the electron beam, the thermal conductivity of the pristine nanorods needs to be considered, as the thermal conductivity of oxides is typically low in comparison to a metal. Additionally, the heat transfer happens through the contact areas between the nanorods, which impedes the thermal conductivity further in comparison to the bulk material, due to rather small contact areas. In a simplified approach the energy resulting from the inelastic scattering of electrons in the material is converted into heat.^57–59^ Considering these two points: the low heat conductivity and the heat induction from the electron beam, we assume that the temperature increases considerably due to electron irradiation.

An additional point, which needs to be taken into consideration, is that vacuum conditions, which are present in SEM and TEM, favour the reduction of oxides.^60,61^ The interplay of the vacuum conditions, the increased temperature, the mobility under the electron beam^47–51^ and the availability of electrons in the system is sufficient for the reduction of Ag-oxide during electron microscopy.

The influence of the temperature as a major factor for the electron beam induced change of morphology is supported by two additional findings. First, the change of morphology was not observed when the particles were deposited on a carbon pad placed onto an SEM stub. In contrast to this, the change was seen quite swiftly on the TEM grid. It can be assumed that the heat conduction of the SEM stub is higher compared to that of the TEM grid, as there is bulk material available that can conduct heat, leading to an increased heat influence of the particles on the TEM grid. Therefore, the heat induction from the electron beam is an influencing factor in the nanorod morphology change. Secondly the difference of the morphology alteration rate between SEM and STEM imaging is important evidence. In the following the stopping power is taken as a dimension for the inelastic interactions and thus the heat transfer of the electrons.^57^ The National Institute of Standards and Technology^62^ states the total stopping power of an electron beam of 200 keV in Cu to be 1.88 MeV cm^2^ g^−1^ and that of 20 keV to be 8.08 MeV cm^2^ g^−1^. Respectively the values for Ag are 1.7 MeV cm^2^ g^−1^ and 7.03 MeV cm^2^ g^−1^. The values of Ag and Cu do not coincide with the true values of the oxide particles, but it is expected that the trend for the stopping power ratio should not deviate. As the SEM probe has a lower acceleration voltage, the interaction cross section gets larger and, thus, more energy is introduced into the material during the SEM imaging process compared to the TEM. Additionally, a larger part of the interaction volume is located within the sample for an SEM probe compared to a STEM probe. In combination with the presumptions made above the heat influence of the SEM probe should also be higher. Thus, the morphology change is expected to proceed faster, which matches with our observations.

We calculate the total dose deployed during the TEM experiment shown in Fig. 4a and c according to Fritsch *et al.*^63^ A beam current of 155 pA, a sample thickness of 100 nm, and a mean beam diameter of 1.76 nm ^64^ (see the ESI†) yield an electron flux density of 3.9 × 10^6^ e^−^ Å^−2^ s^−1^. To convert the electron flux density into a dose rate, we use a mean total stopping power of 1.79 MeV cm^2^ g^−1^ ^62^ for 200 keV electrons using the values stated above, an inelastic mean free path of 100 nm ^65^ for both elements, and a maximum sample thickness of 100 nm (approximately the diameter of the rods in Fig. 4a, assuming a cylindrical geometry). These values give us a dose rate of 2.23 × 10^13^ Gy s^−1^. With a dwell time of 50 μs and 86 frames in total (pixel size: 3.61 nm, frame time: 6.88 s), the maximum dose is calculated to be 9.55 × 10^10^ Gy deployed per pixel over a duration of 592 s.

The same calculation can be done for the SEM part. Here, a beam current of 2 nA and a pixel size of 2.5 nm leads to an electron flux density of 20 × 10^6^ e^−^ Å^−2^ s^−1^. With a mean stopping power of 7.55 MeV cm^2^ g^−1^,^62^ a sample thickness of 100 nm and an averaged inelastic mean free path of 17.6 nm ^66^ for 20 keV, a dose rate of 1.61 × 10^15^ Gy s^−1^ can be calculated. A dwell time of 50 ns yields a dose of 8 × 10^7^ Gy per pixel for one frame.

The differing dose rates for the SEM and STEM investigations (1.61 × 10^15^ Gy s^−1^ and 2.23 × 10^13^ Gy s^−1^, respectively) help to explain the observed artificial decomposition. We see a more rapid decomposition for SEM investigations, than for STEM investigations, which is in line with the dose rates (rate of absorbed inelastic energy of the specimen) being higher for the SEM investigation.

To give an analytical impression of the temperature increase, in the “ESI section: analytical determination of temperature increase”† an estimation of the temperature increase is given, based on ref. 57. The results underline nicely our observed trends: for a TEM substrate in a SEM investigation we determined temperatures of around 90 °C, while for a SEM investigation on a flat bulk substrate, the determined value was half of that on the TEM substrate. For TEM investigations, we calculated temperatures close to atmosphere temperatures. Even though it needs to be highlighted that the determined values are to be seen as a rough estimation, the trend matches with our experimental observations and clearly supports our conclusion that temperature must play a role in the observed decomposition.

#### Electrochemically deployed particles

A change of the microstructure was also observed while imaging the residual nanorods in the TEM. Here the evolution was comparably small. This change was not directly observed with an SEM probe. However, since the surface appears significantly disordered, it cannot be excluded.

The urchin-structure is influenced by electron beam irradiation. SEM studies (Fig. 4, ESI†) show an increasing pattern structure with illumination time. As discussed in the section above, the patterned structure resembles the structure of the chemical pattern of Cu and Ag, as seen in the APT (Fig. 9, ESI†) and STEM-EDXS measurements (Fig. 7, ESI†). During STEM imaging the bright structures increase in size and pores develop inside the urchin-structure.

In summary, interpreting data of nanoparticles including Ag or Ag-oxide requires caution, when electron microscopy is involved in the study. This is not limited to the nanoparticles of this study, but also others, as in general a delicate nature of Ag-containing particles can be assumed. In case of Ag_2_Cu_2_O_3,_ both the pristine and electrochemically-deployed catalyst particles transform under electron beam exposure. Especially the newly evolved urchin-structure proved to be thermodynamically unstable. If one monitors changes during characterizing measurements this transformation of the original state needs to be discussed in sufficient depth.

## Conclusions

In the present study we characterized pristine Ag_2_Cu_2_O_3_ particles and the microstructure that evolved during the electrochemical reaction using SEM, STEM, and APT.

We found that the electron probe has a major influence on the particles. The electron beam triggers a decomposition of the Ag–Cu-oxide, which includes the formation of metallic Ag. The characterization of the particles themselves must be conducted with caution to evaluate the primary structure and not the influence of the electron beam. As the mobility of Ag is known in the literature this cautious interpretation is not only necessary for the Ag_2_Cu_2_O_3_ catalyst, but for other Ag catalyst particles as well.

The investigation of the electrochemically deployed nanorods with near-atomic resolution methods revealed the formation of a three-dimensional Cu–K microstructure, the urchin-structures. This significantly reduces the amount of Cu participating in the reduction of CO_2_. Additionally, the incorporation of K into the microstructure confirms the interaction of K atoms in the catalytic process. K is also incorporated in the structures' interior and its presence is not limited to the surface. This verifies the K-electrolyte to be an additional factor that needs to be considered for the interpretation of the catalytic reaction.

## Conflicts of interest

The authors declare none.

## Supplementary Material

NA-OLF-D5NA00328H-s001

## Data Availability

The data supporting this article have been included as part of the ESI.† Data for this article, including Atom Probe Tomography datasets, will be available at zenodo at https://doi.org/10.5281/zenodo.15045904.
