# Supplementary material for: Understanding the degradation of Ag2Cu2O3 electrocatalysts for CO2 reduction
Source: Nanoscale Adv. 2025 Aug 14;7(19):6005–16. doi: 10.1039/d5na00328h (PMC12352627; doi:10.1039/d5na00328h)
Supplement: NA-007-D5NA00328H-s001 [file NA-007-D5NA00328H-s001.pdf]

Supplementary Information (SI) for Nanoscale Advances.
This journal is © The Royal Society of Chemistry 2025

Supplementary Information

Understanding the degradation of $Ag_2Cu_2O_3$ electrocatalysts for CO_2 reduction

N. Vorlaufer[a], J. Josten[a], A. Hutzler[b], C. A. Macauley[a,c], N. Martić[d], M. Weiser[a], G. Schmid[d], K. J. J. Mayrhofer[b], P. Felfer[a]

[a]Institute I, Materials Science & Engineering Department, Friedrich-Alexander-Universität, Erlangen-Nürnberg (FAU), Martensstraße 5, 91058, Erlangen, Germany

[b]Helmholtz Institute Erlangen-Nürnberg for Renewable Energy (HI ERN), Egerlandstraße 3, 91058, Erlangen, Germany

[c]Interdisciplinary Center for Nanostructured Films (IZNF), Cauerstraße 3, 91058, Erlangen, Germany

[d]GmbH Siemens Energy Global GmbH & Co. KG, SE TI SES PRM CT AEM, Freyeslebenstr. 1, 91058 Erlangen, Germany..

* Corresponding author characterization: nora.vorlaufer@fau.de

Corresponding author catalyst: nemanja.martic@siemens-energy.com

SI Figure 1

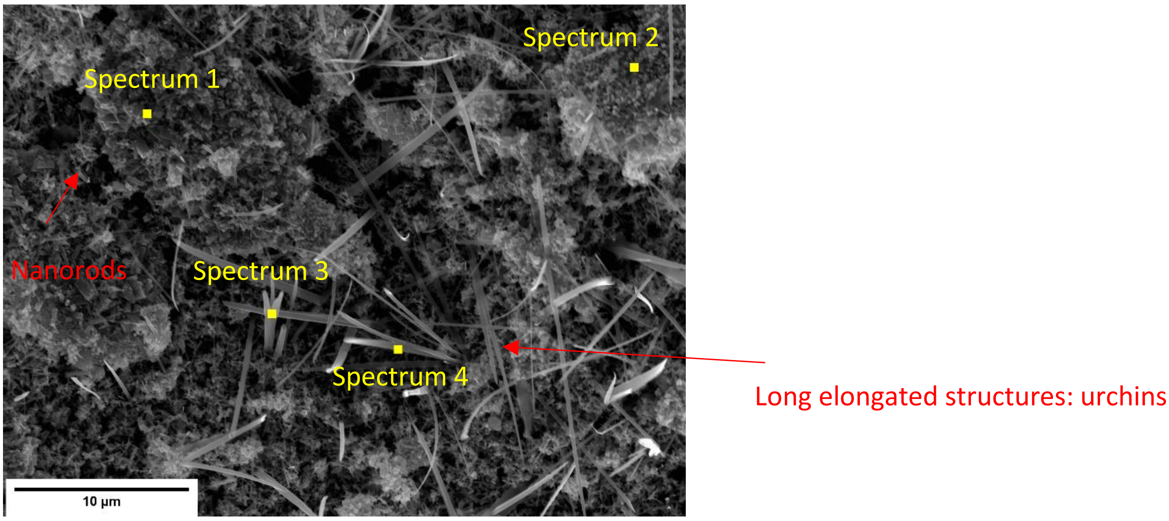

	Spectrum 1 - nanorods	Spectrum 2 - nanorods	Spectrum 3 - urchin	Spectrum 4 - urchin
Cu in at %	40,5	33,3	42,7	46,1
Ag in at %	55,7	62,1	36,5	38,3
K in at %	3,8	4,6	20,8	15,6

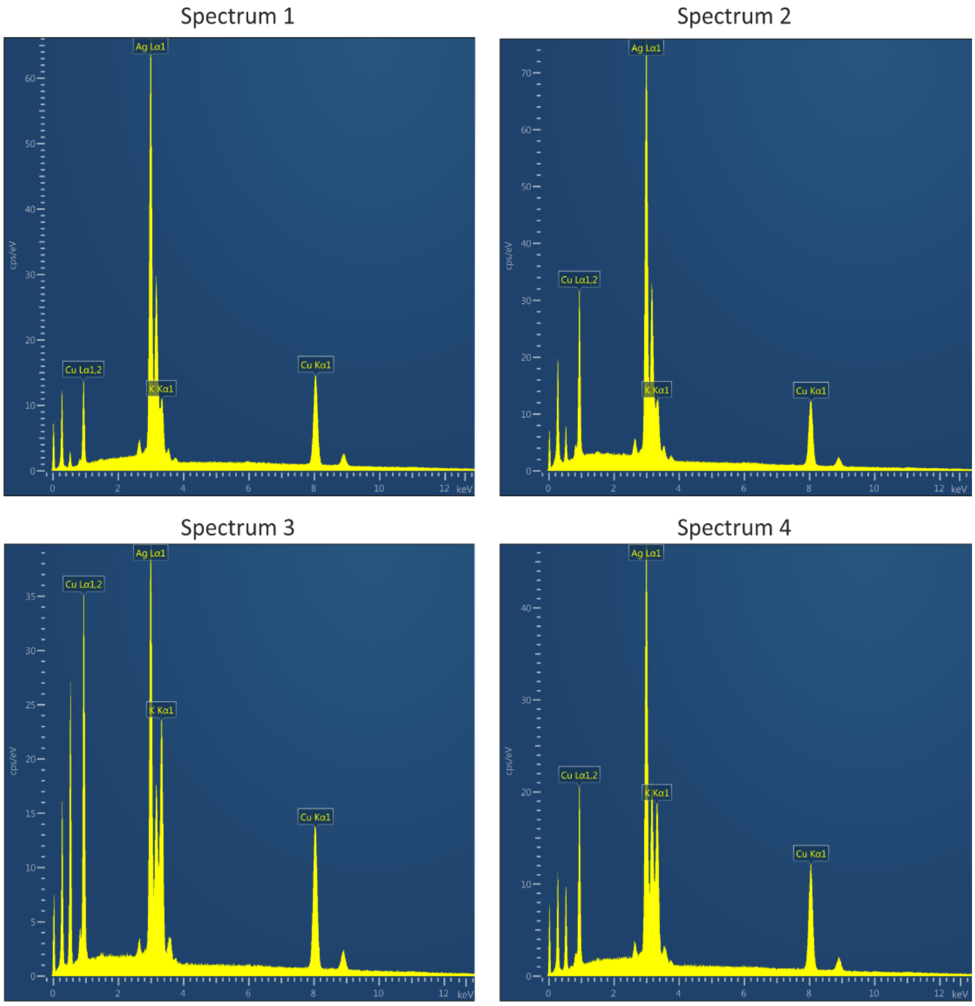

Si Figure 1: SEM image and corresponding EDX spectra. In the SEM image a nanorod-region and an urchin region are highlighted with arrows. The composition of spectra 1 and 2 (region of residue nanorods) and spectra 3 and 4 (urchin-structures) indicate a higher Cu content of the urchin-structures.

SI Figure 2

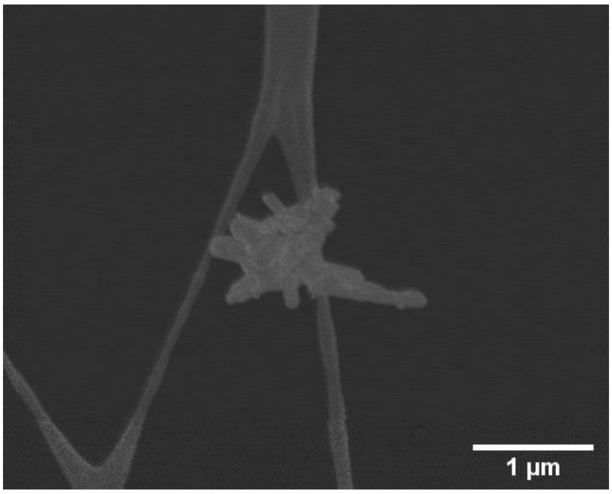

3 kV 500 pA

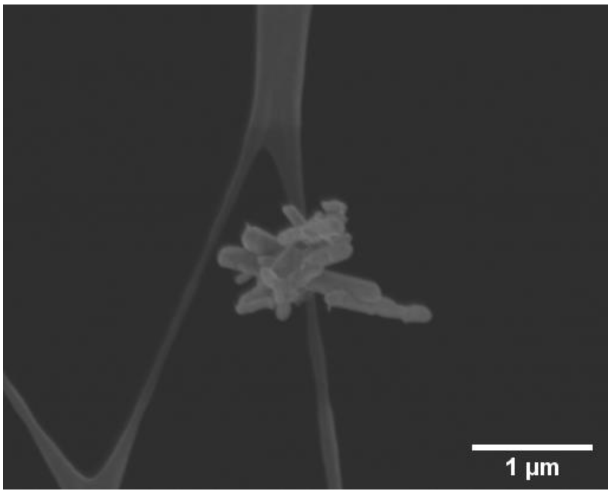

Minimum electron –flux density: 0.95 C/m^2

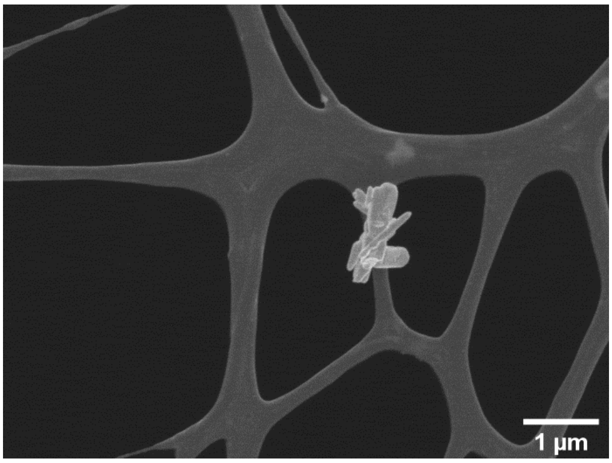

3 kV 3 nA

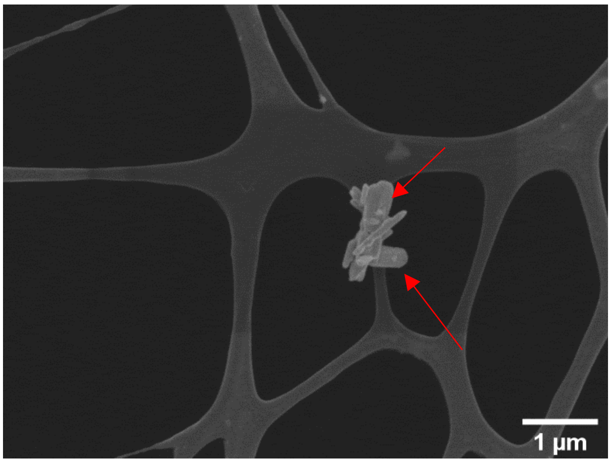

Minimum electron –flux density: 4.91 C/m^2

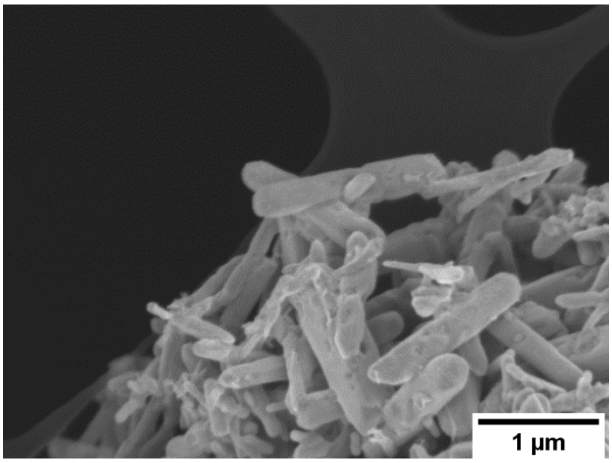

15 kV 3 nA

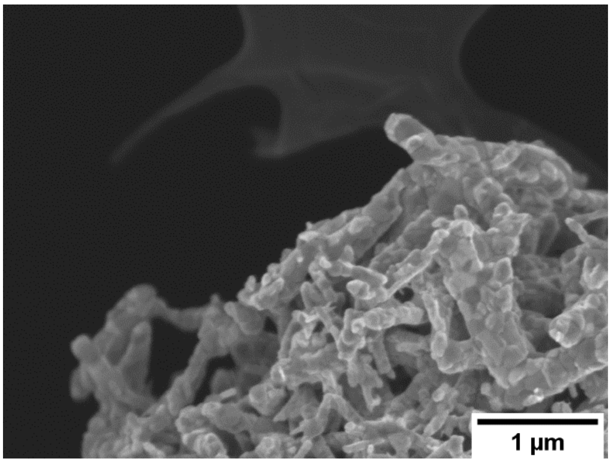

Minimum electron –flux density: 14.67 C/m^2

SI Figure 2: Qualitative illustration of the influence of the SEM electron probe on the pristine particles, which are deposited on a TEM grid. The left column displays the pristine particles after the first image with a minimized electron beam influence. The right column shows the identical particles after repetitive irradiation with the respective electron beam settings. In the voltage regime of the SEM electron beam, a clear dependency on the acceleration voltage is observable. The 15 kV electron beam triggers a more significant change, compared to the 3 kV voltages. Also a higher current affects the particles more clearly. While the particles imaged with 3 kV and 500 pA show no clear morphology change, the 3 kV, 3 nA imaged particles show sphere evolution (highlighted by red arrows). Below the images, a "minimum electron-flux density" for the respective images is stated. This value is indicated as a "minimum" value, as during the imaging and focusing process, which was done nearby, additional electrons, apart from the imaging-electrons, interacted with the particles.

SI Figure 3

150 °C

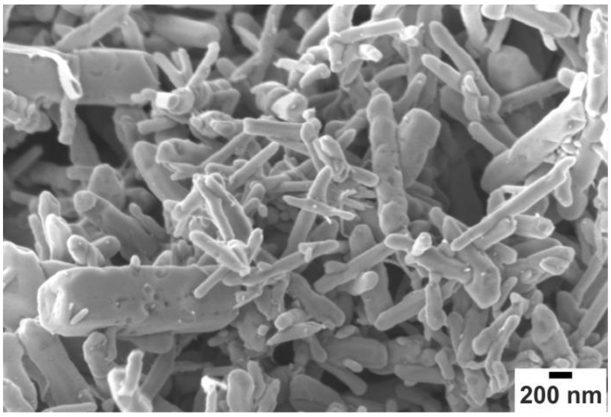

300 °C

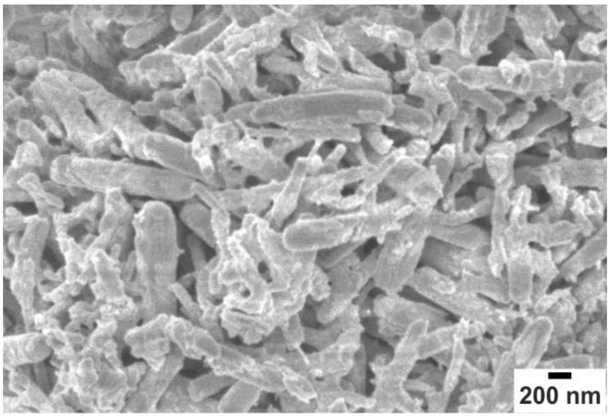

SI Figure 3: Pristine particles after a 150 °C/300 °C heat treatment for 1 h. The heat treatment at 300 °C leads to a distinct morphology change of the particles.

SI Figure 4

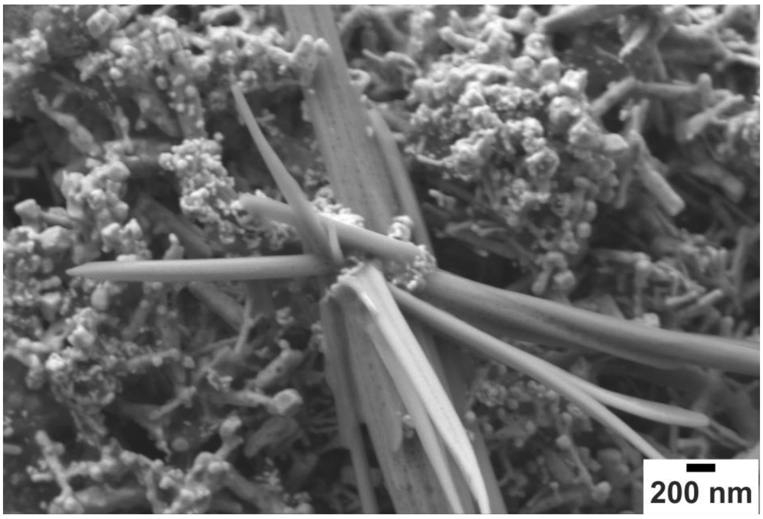

Urchin-structure after one electron beam cycle.

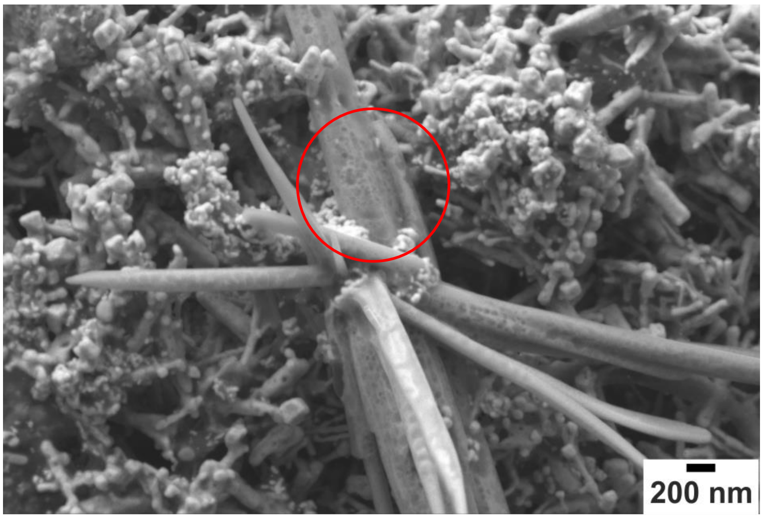

The identical urchin after repetitive irradiation with the electron probe. Exemplary surface changes are highlighted by the red circle.

SI Figure 4: Repetitive irradiation of the urchin-structure with the electron beam, leads to a more pronounced patterned structure on the surface of the urchin.

SI Figure 5

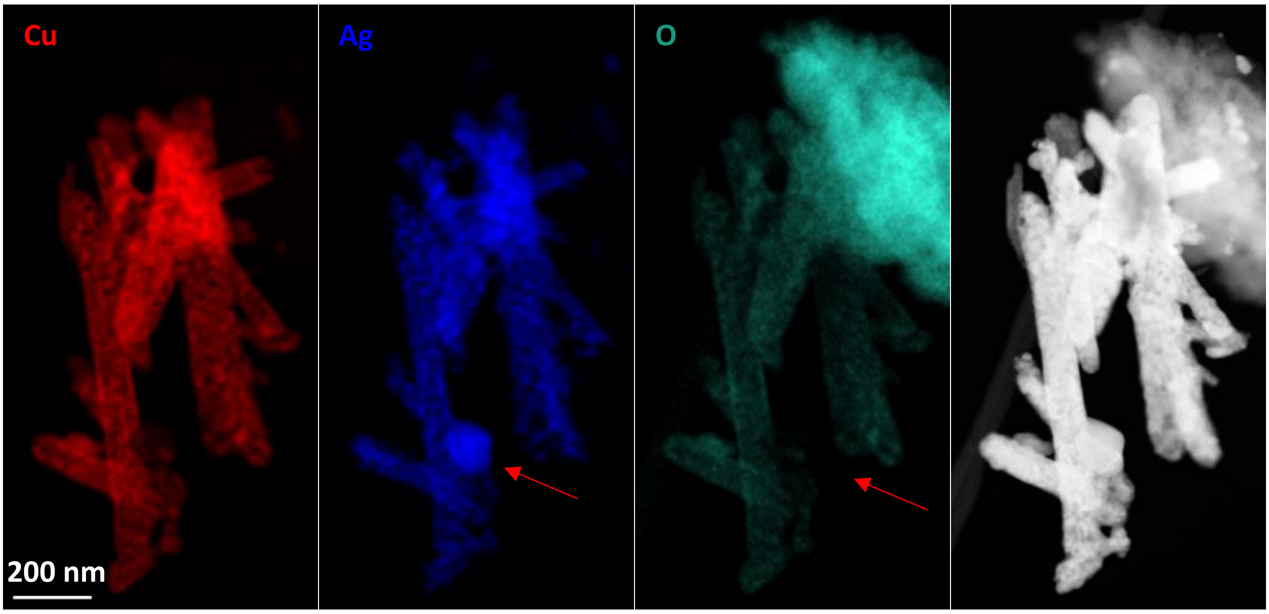

SI Figure 5: Cu, Ag and O EDXS map after electron induced decomposition of a pristine nanorod. The oxygen map also shows a patterned structure. In Ag-rich areas, no residual oxygen is detected. A prominent feature is highlighted with a red arrow.

SI Figure 6

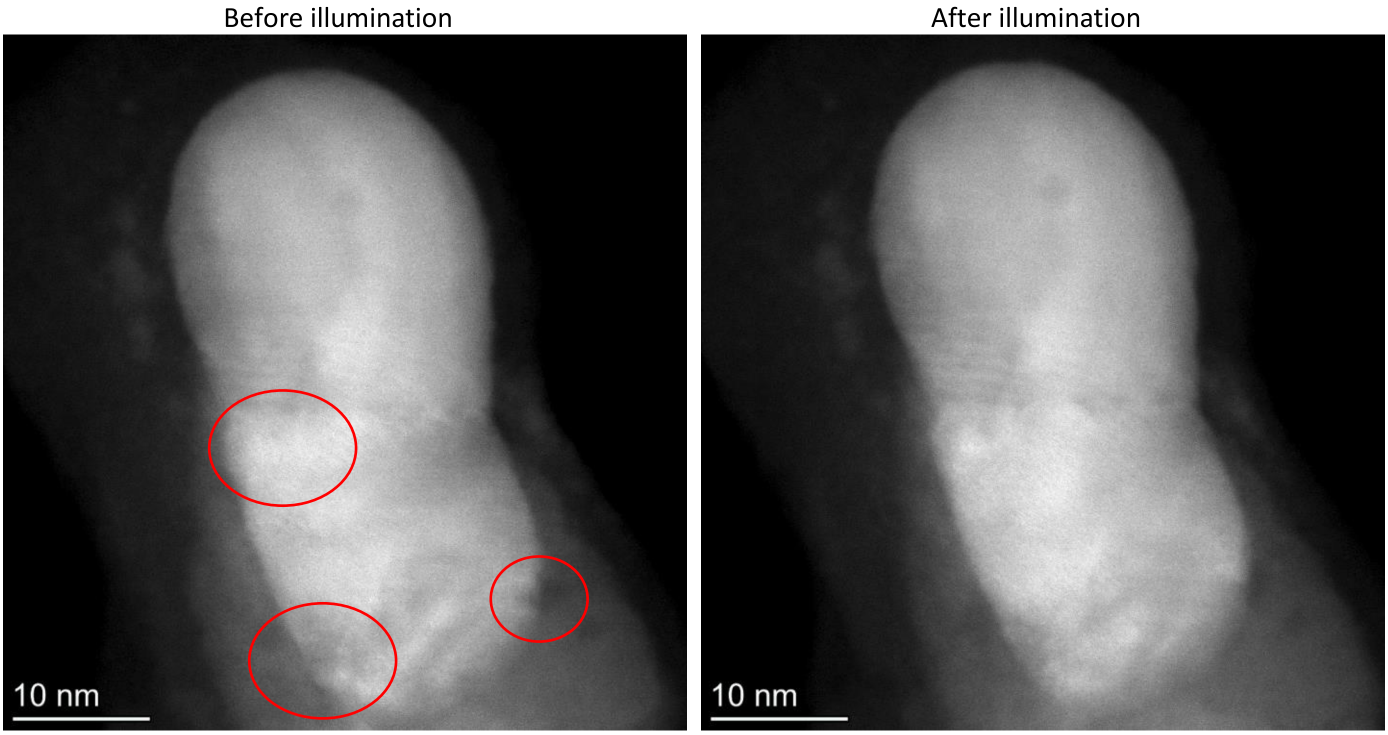

SI Figure 6: Residual nanorod before and after irradiatopn with the TEM electron probe in a TEM. Areas with small change are highlighted with a red circle.

SI Figure 7

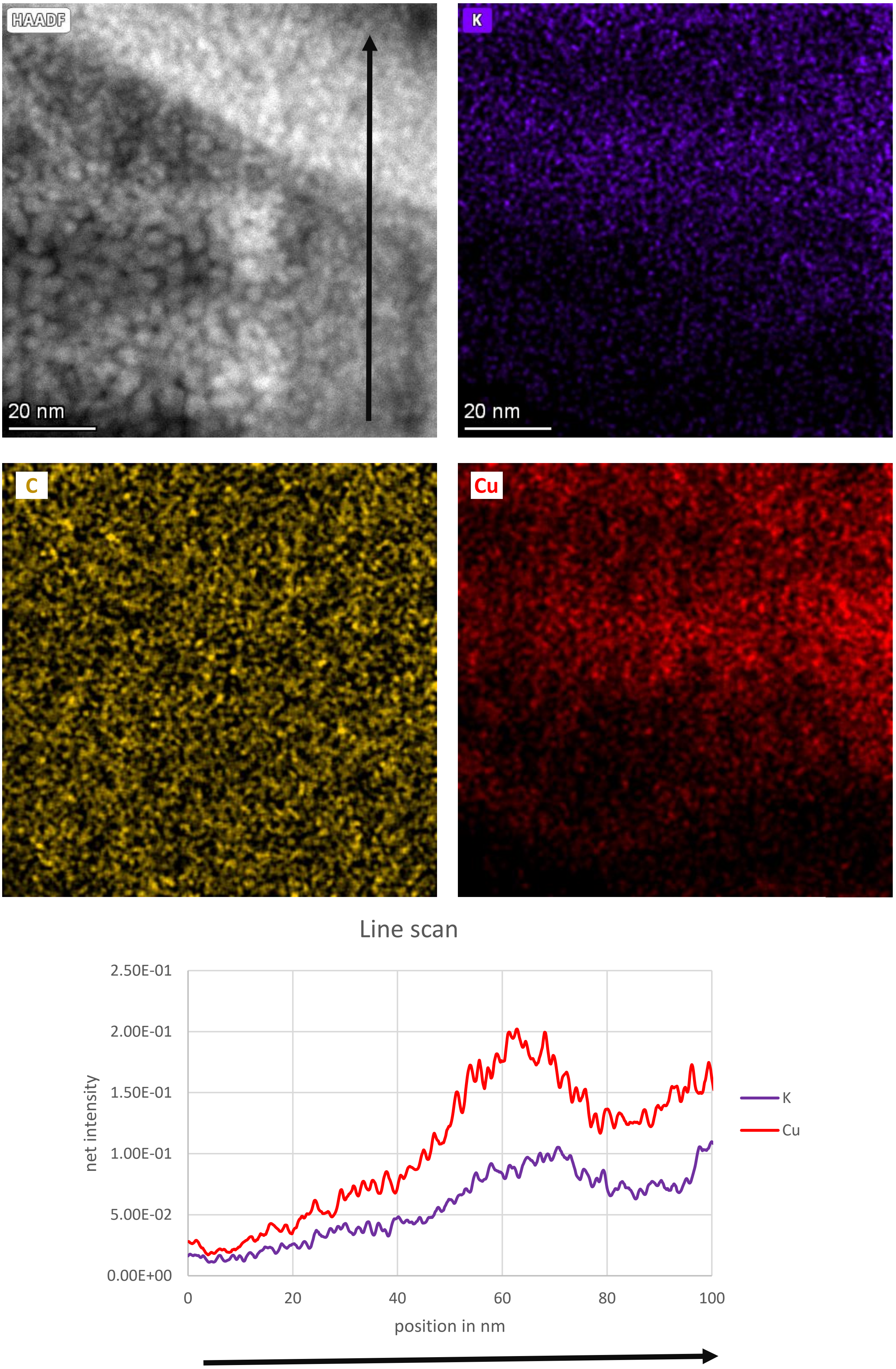

HAADF
20 nm
K
20 nm
C
Cu
Line scan
2.50E-01
2.00E-01
1.50E-01
1.00E-01
5.00E-02
0.00E+00
net intensity
0
20
40
60
80
100
position in nm
K
Cu

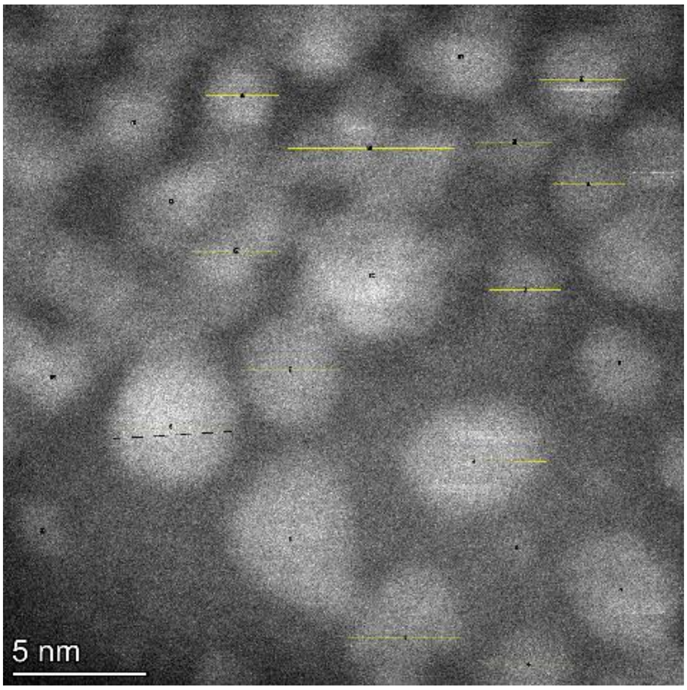

mean value particle diameter: 3,6 ± 1,1 nm

Before intense irradiation

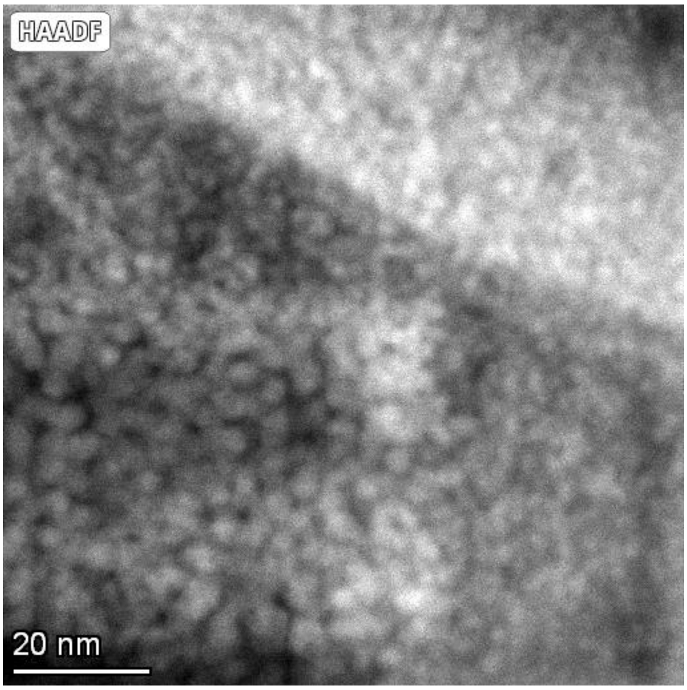

After intense irradiation
(EDXS scan for several minutes)

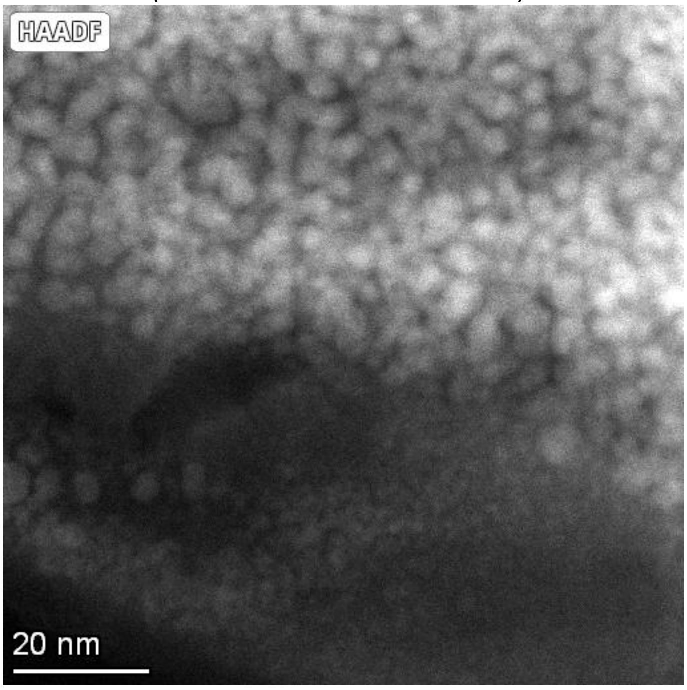

SI Figure 7: Close-up HAADF-STEM investigation of an urchin-structure. HAADF-STEM images reveal the K and Cu content. The white agglomerates inside the urchin usually have a diameter below 4 nm. Size and morphology change during irradiation with the electron probe. Additionally new pores evolve.

SI Figure 8

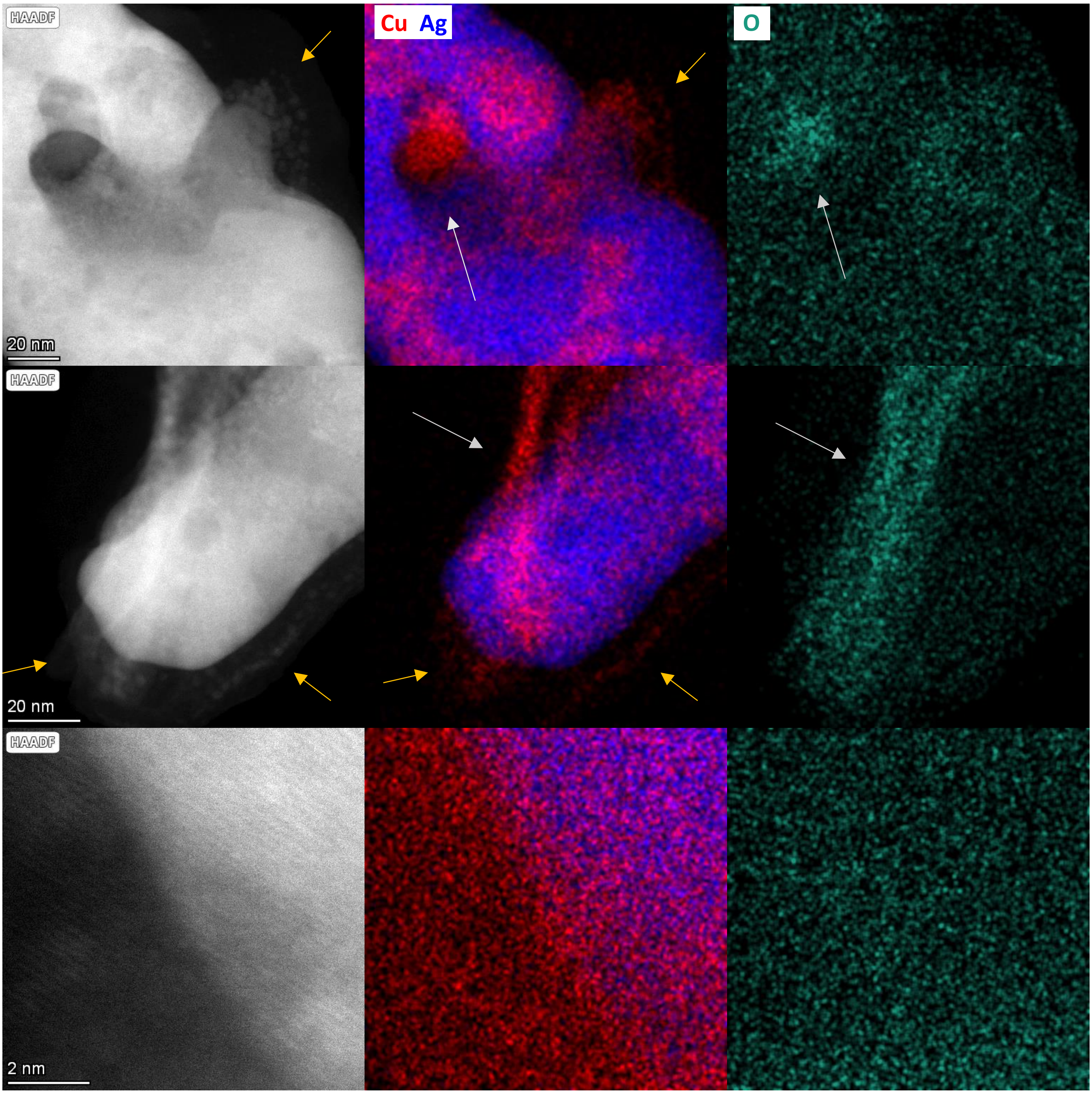

SI Figure 8: A selection of HAADF-STEM images and the corresponding spectrum images of Cu-Ag and O of residual nanorods. The measurement reveals Cu clouds around the residual nanorods, partially covering the surface. The yellow arrows exemplarily highlight the Cu-clouds around the particles. The last image row shows a close-up of the particle-cloud transition at a particles' exterior. The O-map shows oxygen information in the whole region of the particles. Two prominent examples, where Cu and O are enriched in an identical location, are marked with grey arrows. We attribute the oxidized Cu to oxidation in ambient air.

SI Figure 9

Color code of the concentration maps:

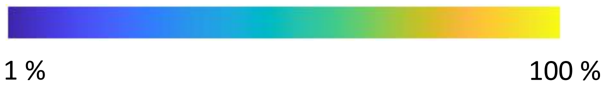

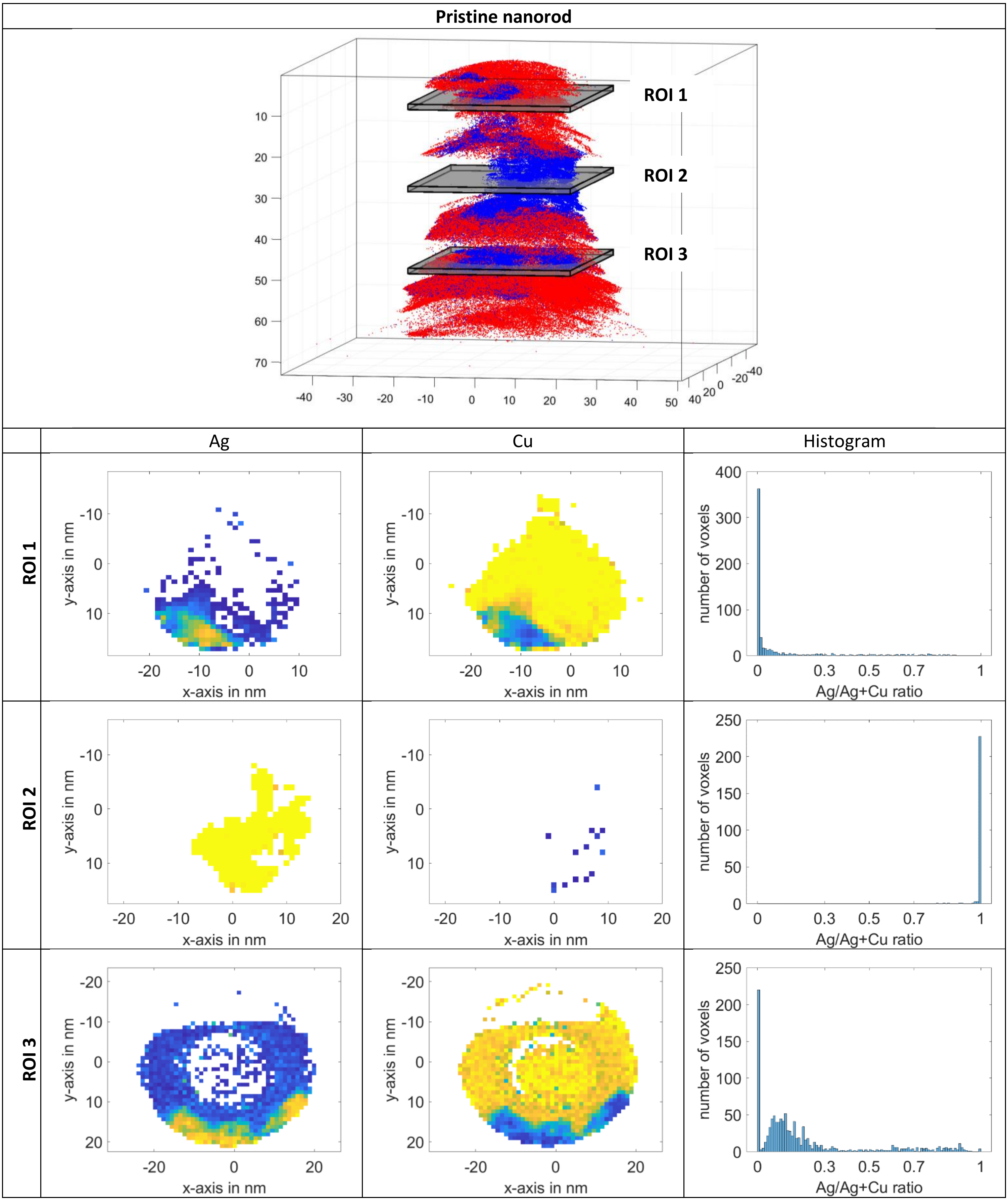

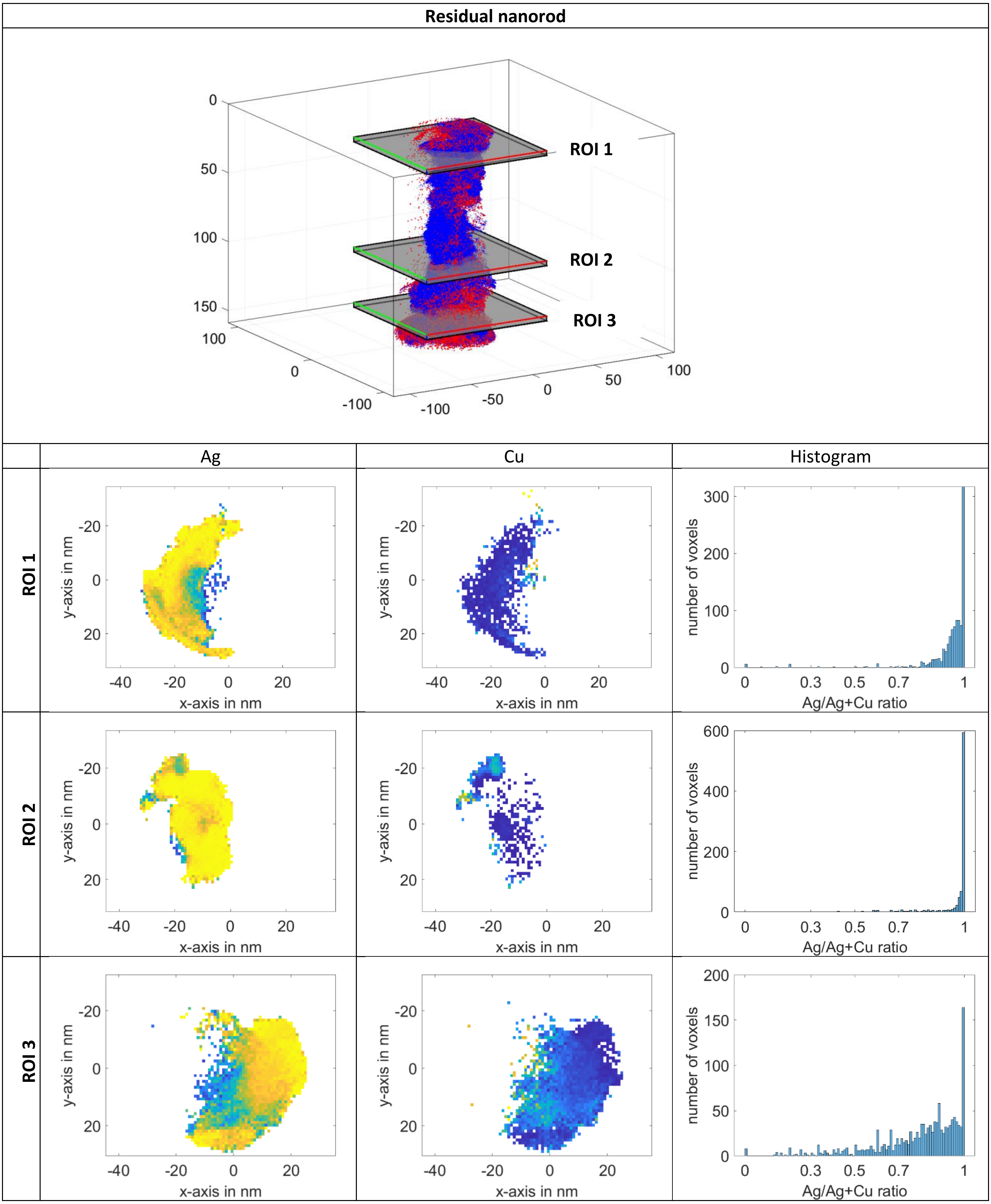

Residual nanorod
0
50
100
150
100
0
-100
-100
-50
0
50
100
ROI 1
ROI 2
ROI 3
Ag
Cu
Histogram
ROI 1
ROI 2
ROI 3
y-axis in nm
x-axis in nm
-20
0
20
-40
-20
0
20
number of voxels
Ag/Ag+Cu ratio
0
0.3
0.5
0.7
1
300
200
100
600
400
200
150
50

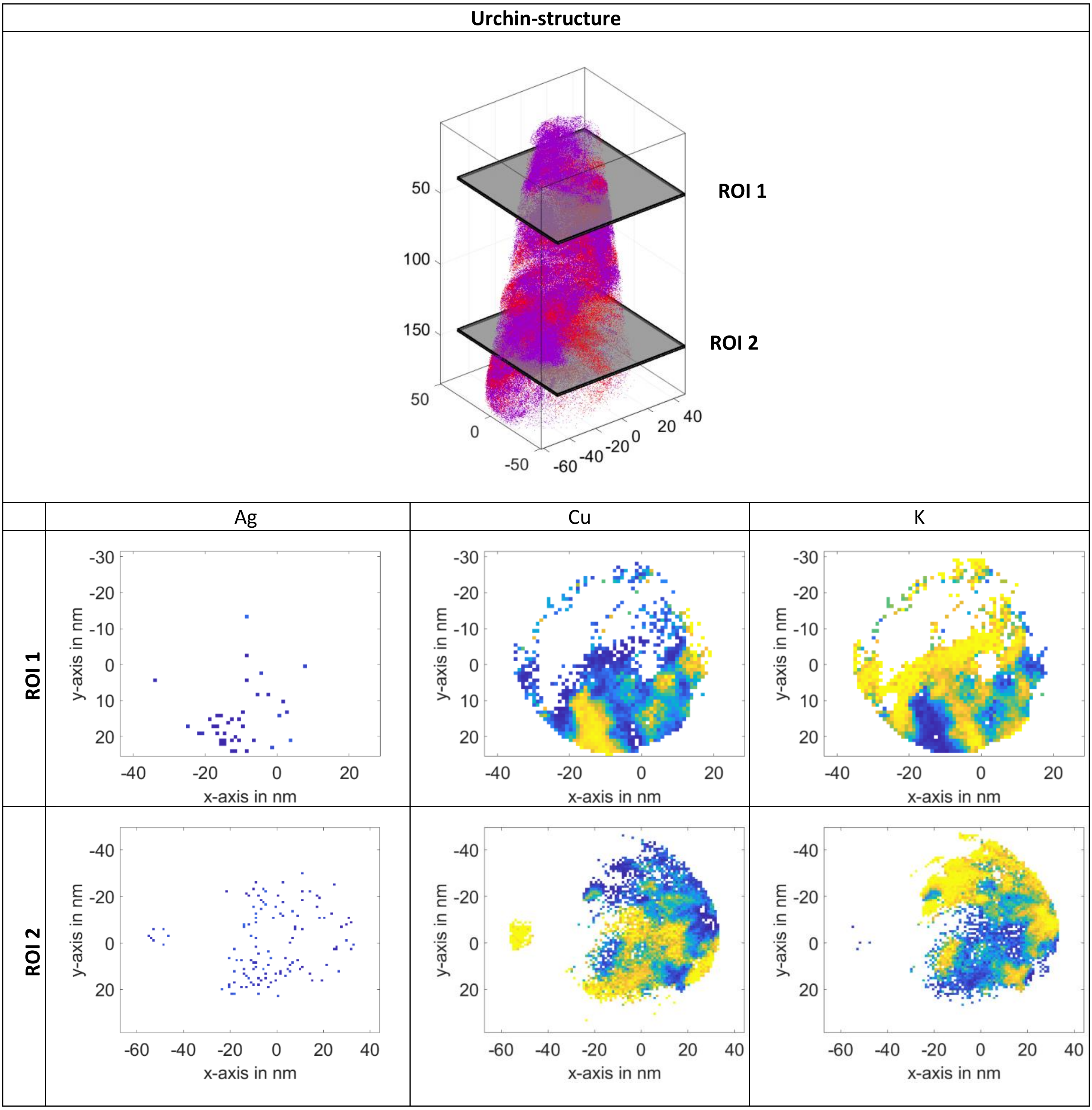

SI Figure 9: Analysis of the APT data of pristine-nanorod, residual-nanorod and urchin structure. For each ROI the 2D-concentration of Ag and Cu, for the urchin-structure additionally the K-concentration, is shown. The histogram is an indication for the main present element and wether the atoms are present in a mixture, or demixed.

SI Figure 10

Urchin-structure on grid

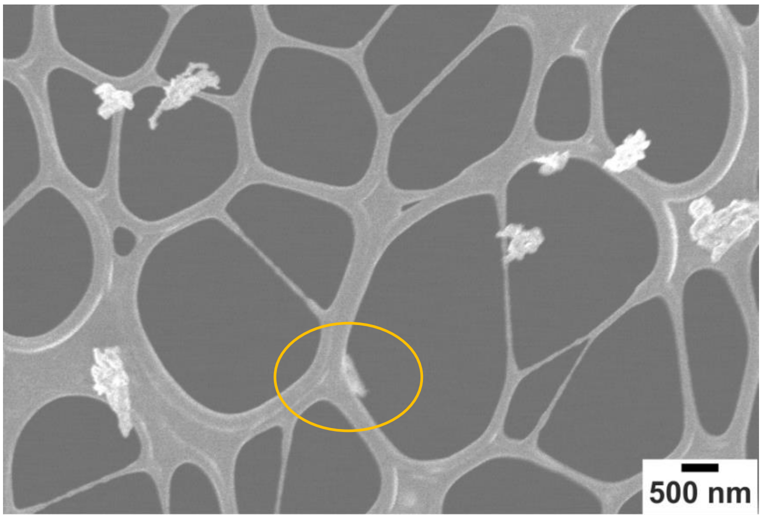

Residual nanorod on grid

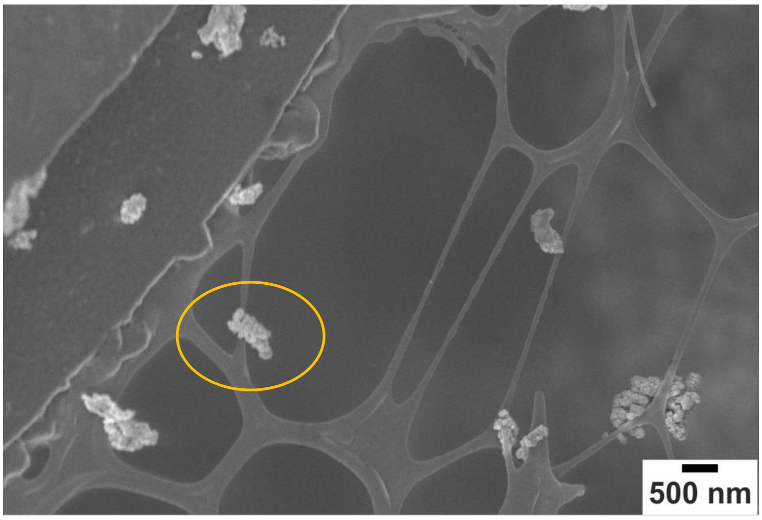

		Ag	Cu	K
**Urchin**	Number of atoms	14276	1168152	2914657
	Atom percent	0.35	28.51	71.14
	Standard deviation	$2.31*10^{-5}$	$1.16*10^{-4}$	$1.77*10^{-4}$
**Residual nanorod**	Number of atoms	9558218	498295	389433
	Atom percent	91.5	4.77	3.72
	Standard deviation	$7.02*10^{-5}$	$5.19*10^{-5}$	$4.63*10^{-5}$

SI Figure 10: SEM images of the picked electrochemical deployed particles for the APT measurement. The table shows the counts of atoms measured for the respective particle in the APT. The given standard deviation was calculated based on [1], with the following formula: standard deviation= $\sqrt{(p_0\,(1-p_0))*((1-Q)/n)}$, with the detector efficiency *Q*, the total number of detected atoms *n* and p_0 the proportion of the detected species' atoms to all detected atoms of the probed volume *n*.

SI Figure 11

Spectrum nanorod pristine

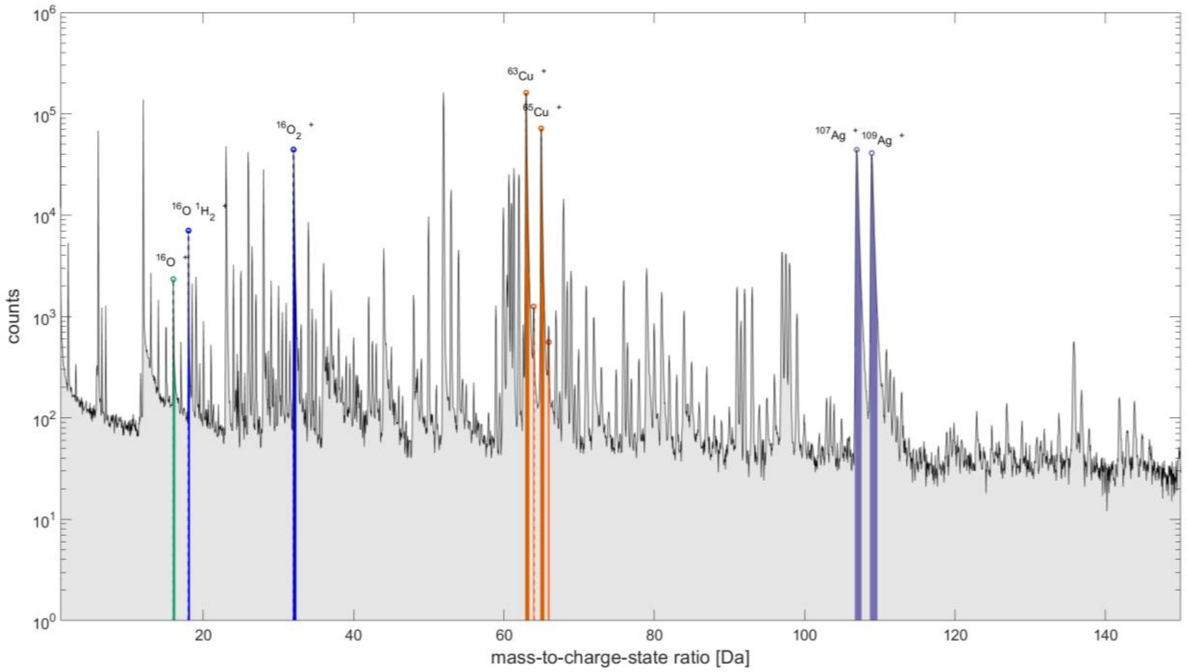

Spectrum urchin

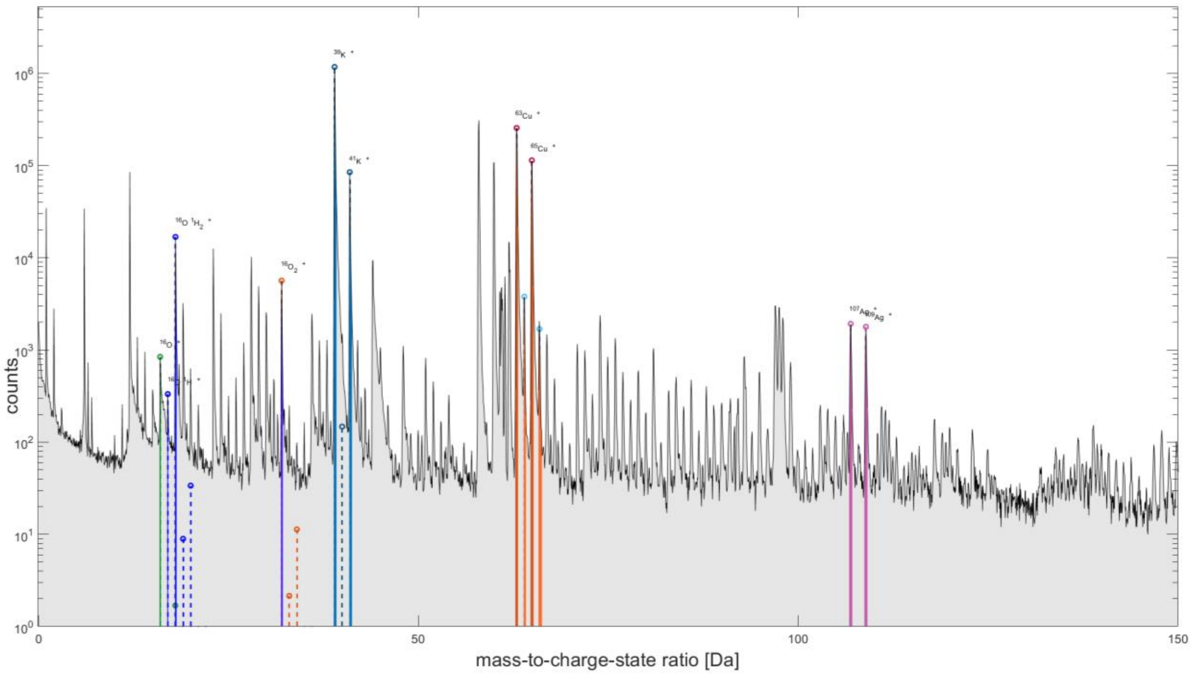

Spectrum residual nanorod

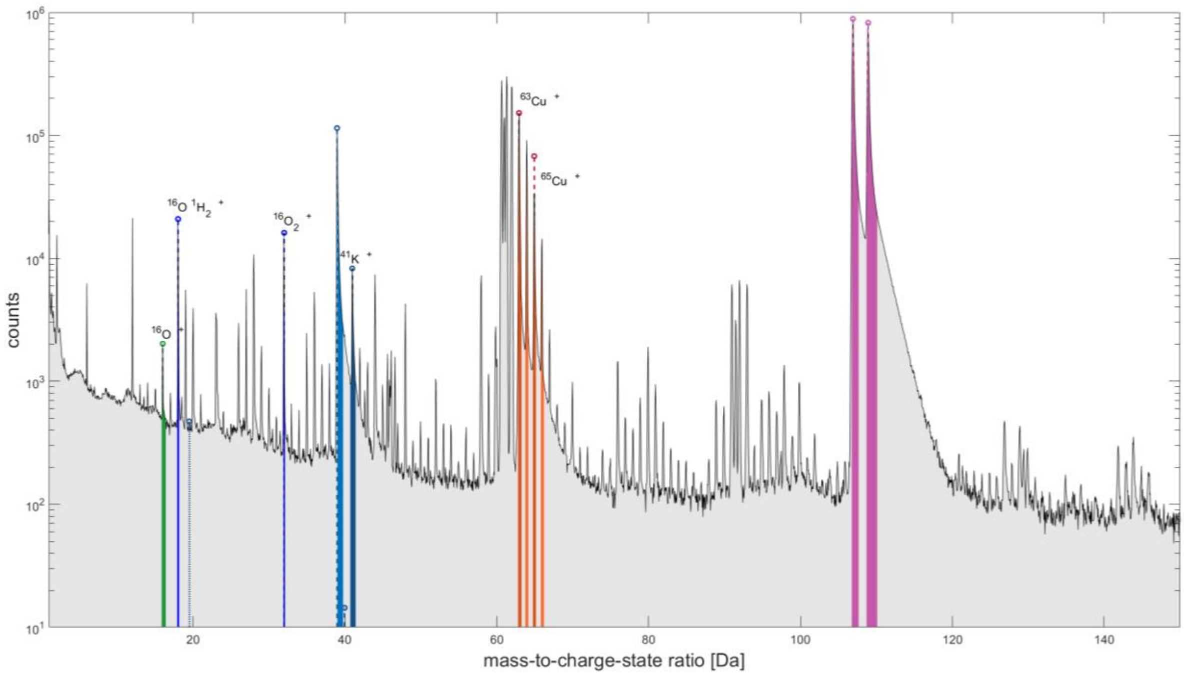

SI Figure 11: Overview of three mass spectra of the APT measurements. To maintain a clear overview, in each spectrum, the peaks of the relevant ions are highlighted: O, K, Cu and Ag. Other main peaks in the spectrum belong to the coating material, which is either Ni or Cr, and the support material W. Especially for the coatings, several oxide peaks were measured, making up the largest part of the residual spectrum.

SI section: beam diameter TEM

A beam diameter of 1.23 nm (full width at tenth maximum) focused on top of the sample, a beam vergence of 10.5 mrad, and a sample thickness of 100 nm yields an exit beam diameter of 2.3 nm according to Körner et al.[2]. With this, the mean beam diameter amounts to (1.23 nm+2.28 nm)/2 = 1.76 nm

SI section: analytical determination of temperature increase

In the following an analytical estimation of the temperature rise during electron beam illumination is given. The estimation is carried out based on the publication from Tivol [3]. In general, this estimation is a qualitative assessment; simulated or numerical approaches are to be preferred.

In the publication, the heat input is calculated by multiplying the stopping power, with the density, the electron flux and the particle volume. It is assumed, that the heat loss takes place due to radiation loss and conductive loss. In our case, the radiation loss is negligible, as it is five order of magnitudes lower than the conductive loss for reasonable temperatures.

In [3] the authors determine the contact area depending on a spherical particle. Deviating from this, nanorods are imaged in this work. So, we assume the contact area to be the base area of the nanorod. For imaging on a TEM grid, we assume that the contact area is one third of the nanorods base area, for imaging on a flat substrate the whole base area is taken as contact area between substrate and nanorod.

As there are no values for the stopping power of Cu-oxide/Ag-oxide, which are known to the authors, the calculations are done based on the respective values of the metals. To calculate the heat input based on [3] the following data are used:

The other utilized values are:

Beam current SEM [nA]	2
Beam current TEM [nA]	0.041
pixel size SEM [nm]	3
pixel size TEM [nm]	2.24

	Density[4] [kg/m³]	thermal conductivity[5] [W/(cm*K)]	stopping power 20 keV[4] [MeV*cm^2/g]	stopping power 200 keV[4] [MeV*cm^2/g]
Ag	10503	429	7.03	1.7
Cu	8954	400	8.08	1.88

The substrate/atmosphere temperature were assumed to be 300 K, the average nanorod was assumed to have a height of 1000 nm and a diameter of 300 nm.

Using this data, a temperature of 84 °C for Ag in the SEM was calculated, for Cu the value is 87 °C. Here, the particles are imaged on a TEM grid. For all TEM calculations, the values were in the range of ~28 °C. For imaging on a flat bulk substrate, the calculated temperature is 46 °C and 47 °C for Ag and Cu respectively. So, the difference of the values for the TEM grid and the flat bulk substrate (both imaged in the SEM) in this approach can mainly be attributed to the different contact areas of the nanorod with the substrate, which is a major influence factor of the heat conduction out of the particle.

The calculated values are a rough, qualitative assessment, however they highlight the assumed relationships: the observed decomposition in the TEM was lower, than for the SEM. This is in good agreement with the calculated temperature rise.

Also, the nanorod temperature during imaging on a flat bulk substrate is lower, compared to imaging on a TEM grid. This matches our observation, that no artificial decomposition was observed on a SEM stub, but on a TEM grid.

References

(1) Danoix, F.; Grancher, G.; Bostel, A.; Blavette, D. Standard Deviations of Composition Measurements in Atom Probe Analyses—Part II: 3D Atom Probe. *Ultramicroscopy* **2007**, *107* (9), 739–743. https://doi.org/10.1016/j.ultramic.2007.02.005.

(2) Körner, A.; Fritsch, B.; Morales, A. L.; Malgaretti, P.; Hutzler, A. Panta Rhei - Tuning Silver Nanostructure Evolution with Flow and Radiolysis in Liquid Phase STEM. *Nano Today* **2025**, *61*, 102575. https://doi.org/10.1016/j.nantod.2024.102575.

(3) Tivol, W. F. How to Calculate the Temperature Rise Due to Beam Heating. *Microsc. Today* **1999**, *7* (7), 24–27. https://doi.org/10.1017/S1551929500064774.

(4) Seltzer, S. Stopping-Powers and Range Tables for Electrons, Protons, and Helium Ions, NIST Standard Reference Database 124, 1993. https://doi.org/10.18434/T4NC7P.

(5) Ho, C. Y.; Powell, R. W.; Liley, P. E. Thermal Conductivity of the Elements. *J. Phys. Chem. Ref. Data* **1972**, *1* (2), 279–421. https://doi.org/10.1063/1.3253100.
